# Metabolomic Markers of Phthalate Exposure in Plasma and Urine of Pregnant Women

**DOI:** 10.3389/fpubh.2018.00298

**Published:** 2018-10-22

**Authors:** Michael Zhou, Breanna Ford, Douglas Lee, Gwen Tindula, Karen Huen, Vy Tran, Asa Bradman, Robert Gunier, Brenda Eskenazi, Daniel K. Nomura, Nina Holland

**Affiliations:** ^1^School of Public Health, Center for Environmental Research and Children's Health, University of California, Berkeley, Berkeley, CA, United States; ^2^Departments of Chemistry, Molecular and Cell Biology, and Nutritional Sciences and Toxicology, University of California, Berkeley, Berkeley, CA, United States; ^3^Omic Insight, LLC, Durham, NC, United States

**Keywords:** targeted metabolomics, endocrine disruptors, phthalates, *in utero* exposure, inflammation, pregnancy

## Abstract

Phthalates are known endocrine disruptors and found in almost all people with several associated adverse health outcomes reported in humans and animal models. Limited data are available on the relationship between exposure to endocrine disrupting chemicals and the human metabolome. We examined the relationship of metabolomic profiles in plasma and urine of 115 pregnant women with eleven urine phthalate metabolites measured at 26 weeks of gestation to identify potential biomarkers and relevant pathways. Targeted metabolomics was performed by selected reaction monitoring liquid chromatography and triple quadrupole mass spectrometry to measure 415 metabolites in plasma and 151 metabolites in urine samples. We have chosen metabolites with the best defined peaks for more detailed analysis (138 in plasma and 40 in urine). Relationship between urine phthalate metabolites and concurrent metabolomic markers in plasma and urine suggested potential involvement of diverse pathways including lipid, steroid, and nucleic acid metabolism and enhanced inflammatory response. Most of the correlations were positive for both urine and plasma, and further confirmed by regression and PCA analysis. However, after the FDR adjustment for multiple comparisons, only 9 urine associations remained statistically significant (*q*-values 0.0001–0.0451), including Nicotinamide mononucleotide, Cysteine T2, Cystine, and L-Aspartic acid. Additionally, we found negative associations of maternal pre-pregnancy body mass index (BMI) with more than 20 metabolomic markers related to lipid and amino-acid metabolism and inflammation pathways in plasma (*p* = 0.01–0.0004), while Mevalonic acid was positively associated (*p* = 0.009). Nicotinic acid, the only significant metabolite in urine, had a positive association with maternal BMI (*p* = 0.002). In summary, when evaluated in the context of metabolic pathways, the findings suggest enhanced lipid biogenesis, inflammation and altered nucleic acid metabolism in association with higher phthalate levels. These results provide new insights into the relationship between phthalates, common in most human populations, and metabolomics, a novel approach to exposure and health biomonitoring.

## Introduction

Environmental metabolomics, a newly emerging approach to characterize the entirety of metabolites found in biological specimens to obtain insight into the relationship with expression, epigenetics, and various exposures, is attracting increasing attention (1–3). Metabolomics is a complex dynamic field of research that explores thousands of compounds in different body fluids that are constantly changing due to the influence of the environment, disease, life style factors and microbiome (4). These scientific advancements are made possible by recent technological developments enabling comprehensive simultaneous analysis of a large number of molecules in minute volumes of biological specimens, permitting evaluation of chemical modifications in the body that occur due to exposure or in relation to health outcomes (5). A number of targeted and untargeted approaches have been explored in this rapidly developing field of research. However, for human population studies focused on specific health outcomes or exposures, targeted metabolomics that assesses hundreds rather than tens of thousands of compounds may be more appropriate.

Mass spectrometry (MS) and nuclear magnetic resonance (NMR) have emerged as the two popular analytic methodologies for metabolomic research. NMR is often used for untargeted studies where the main focus is profiling the entire complement of metabolites in a sample often reaching tens of thousands of compounds but sometimes with low sensitivity (6). MS is used more commonly for targeted profiling when specific molecules are characterized and the method can be optimized to minimize interference with other metabolites (4, 7).

Phthalates are often added to industrial and consumer products as solubilizing agents and to increase product flexibility (8). Since they are not chemically linked to their substrates, they have been found in household air, dust, and various foods and drinks. Phthalates are ubiquitous in the environment and recent biomonitoring studies indicate widespread exposure in U.S. populations (9) and developing embryos can also be exposed to phthalates. The majority of pregnant women were found to have detectable phthalate concentrations in their urine (10). Early life exposure to phthalates has been related to adverse health outcomes in animals and humans, indicating a connection to the fetal origins of disease (11–14).

Animal studies suggest that applying metabolomic approaches to prenatal phthalate exposure may inform research on the mechanisms linking developmental exposure to phthalates with detrimental health effects in humans (15, 16). Experimental results in rodents have shown a dose dependent relationship of metabolomics markers with early life exposure to phthalates in a sex specific manner (17–20).

To date, data on the relationship of phthalate exposure with the human metabolome is limited. In a study of Chinese men with phthalate exposure, elevated oxidative stress and fatty acid oxidation, as well as changes to the urea cycle, tryptophan and phenylalanine metabolism, were observed (21). Two other studies have described metabolomic markers in pregnant women using a targeted approach in relation to obesity (22) and preterm birth (23), but they did not examine the metabolome in relation to environmental toxicants in general or to endocrine disruptors such as phthalates, specifically.

Previously, we characterized concentrations of eleven phthalate metabolites in urine samples from pregnant women (11). The focus of the current study is to explore the relationship between metabolomic compounds found in plasma and urine at the 26th week of gestation with concentrations of phthalate urine metabolites measured at the same time, and to characterize associations with maternal pre-pregnancy BMI.

## Materials and methods

### Maternal demographics and sample characteristics

Plasma and urine used for this study were randomly selected convenience samples collected at 26.4 (±3.2; 20–36) weeks gestation from pregnant women (*N* = 115) from the Center for the Health Assessment of Mothers and Children of Salinas (CHAMACOS) cohort (24). The subset included in this study did not differ in demographic and exposure characteristics, and it was representative of all pregnant women enrolled in 1999–2000. At the time of enrollment, CHAMACOS mothers were at least 18 years of age, eligible for low income health insurance, receiving prenatal care at one of several participating clinics, and had experienced less than 20 weeks gestation. Urine and plasma samples were aliquoted, barcoded, and stored in −80°C freezers at the UC Berkeley School of Public Health. Samples were transferred to the Nomura Research Group on dry ice and stored at −80°C until targeted metabolomic analysis. Women included in this study previously had their phthalate exposure assessed based on concentrations of eleven urine metabolites (12). CHAMACOS study protocols were approved by the University of California, Berkeley Committee for Protection of Human Subjects. All women provided written informed consent at the time of enrollment.

### Phthalate metabolite concentrations

Concentrations of eleven urine phthalate metabolites were characterized by solid phase extraction coupled with isotope dilution high-performance liquid chromatography-tandem mass spectrometry (25), as previously described (11, 12). The metabolites assessed included three LMW metabolites [MEP, mono-n-butyl phthalate (MBP), mono-isobutyl phthalate (MiBP)], four DEHP metabolites [mono(2-ethylhexyl) phthalate (MEHP), mono(2-ethyl-5-hydroxyhexyl) phthalate (MEHHP), mono(2-ethyl-5-oxohexyl) phthalate (MEOHP), mono(2-ethyl-5-carboxypentyl) phthalate (MECPP)], and four high molecular weight (HMW) metabolites (monobenzyl phthalate (MBzP), mono(3-carboxypropyl) phthalate (MCPP), monocarboxyoctyl phthalate (MCOP), monocarboxynonyl phthalate (MCNP)]. In order to ensure quality control, laboratory and field blanks, calibration standards, and spiked controls with low and high concentrations were incorporated into the experimental runs.

Summary measurements of the LMW, HMW, and DEHP metabolites in μg/L were generated by dividing the molar concentrations of the metabolites by an average molecular weight for the group. Urinary phthalate concentrations were log transformed and specific gravity adjusted for further analyses.

### Metabolomic profiling

Plasma and urine samples from the 115 women were analyzed in duplicates. Urine samples were parallel aliquots (that did not undergo previous freeze-thaw) of the same urines that were used for the measurement of the concentrations of eleven phthalate metabolites as noted above (11, 12).

The metabolomics profiles were assessed by selected reaction monitoring liquid chromatography (LC) and triple quadrupole mass spectrometry (MS). The QQQ LC-MS/MS targeted method was chosen as it has been previously validated in Nomura laboratory to measure the abundance of several hundred representative polar and nonpolar metabolites (26). The metabolites analyzed are found in functionally diverse metabolic pathways. Of particular interest to us were pathways that are likely to be affected by phthalate exposure, specifically those induced by MEHP-mediated PPARγ activation, oxidative stress and chronic inflammation pathways that play an important role in mechanisms of obesity (27, 28).

Nonpolar lipid metabolites were extracted from 30 μL serum or urine in 3 ml of 2:1 chloroform: methanol and 1 ml of PBS with inclusion of internal standards C12 monoalkylglycerol ether (MAGE) (10 nmol, Santa Cruz Biotechnology) and pentadecanoic acid (10 nmol, Sigma-Aldrich). Organic and aqueous layers were separated by centrifugation at 1000 × *g* for 5 min and the organic layer was collected, dried under a stream of N_2_ and dissolved in 120 μl chloroform. Polar metabolites were extracted from 30 μL serum or urine in 160 μL of 1:1 (ACN:MeOH) with inclusion of internal standard D^3^N^15^ serine (50 nM, Cambridge Isotope Laboratories, Inc., #DNLM-6863). Urine specimens were vortexed, spun down at 21,000 × g for 10 min and the supernatant was used for experiments. All samples were stored at −80°C until analysis at which time they were thawed, vortexed and placed in an auto-sampler for no more than 24 h prior to mass spec injection.

Metabolomic analysis was performed on an Agilent 6430 by QQQ LC-MS/MS (Agilent Technologies, Santa Clara, California) (29). The capillary voltage was set to 3.0 kV, and the fragmentor voltage was set to 100 V. The drying gas temperature was 350°C with a flow rate of 10.l/min, and the nebulizer pressure was 35 psi. Metabolites were identified by SRM of the transition from precursor to product ions at associated optimized collision energies and retention times as previously described (30). Metabolomic markers were quantified by measuring the area under the curve. Plasma nonpolar positive metabolites were normalized to C12 MAGE, plasma nonpolar negative metabolites were normalized to Pentadecanoic acid, and all plasma polar and urine metabolites were normalized to D^3^N^15^ Serine. Quality assurance and control measures comprised laboratory and field blanks, internal standards. Additionally, incorporated repeat samples showed good reproducibility (CVs ≤ 3–15%).

### Statistical analysis

We first computed descriptive statistics for metabolites to examine their distributions. Spearman correlation coefficients were calculated to determine relationships between phthalate metabolite concentrations and metabolomics profiles. Using these correlation coefficients, we generated heat maps to show the most prominent (*r* > 0.3) positive and negative associations graphically.

We also generated regression models to assess the relationship between concurrent prenatal phthalate levels and metabolomic profiles in pregnant women, which were both log transformed. Separate models were run for the plasma and urine metabolites. In order to account for urinary dilution, plasma models were examined in association with specific gravity adjusted phthalate metabolite levels. Covariates in the plasma and urine regressions included parity (binary; 0 or ≥1) and maternal pre-pregnancy BMI (continuous), since these variables were previously associated with urinary phthalate metabolites in the CHAMACOS cohort (11). After observing significant associations between phthalate metabolites and urine metabolomic compounds, we ran similar regression models using principal components derived from urine metabolomic compounds as the outcome. 16 of the 40 total principal components explained 95% of the variance and were included in the analyses. Additionally, we used regression models to assess the relationship between pre-pregnancy maternal body mass index (BMI) and metabolomics compounds. Urinary metabolites were adjusted for specific gravity (31).

All analyses were performed using STATA 12.0 (StataCorp, College Station, TX) and R 3.3.0 (R Foundation for Statistical Computing, Vienna, Austria) (32, 33).

### Pathway identification

Although correlation analysis used in this study treat the metabolites as if they are independent variables, in fact they are often related by a common metabolic pathway. Alternatively, a single metabolite can participate in several pathways. We used multiple online tools including MetaboAnalyst (34, 35) (v3.0), Impala (36, 37) (v.9.0) and the KEGG Atlas of metabolic pathways (38) to explore which metabolic pathways associated with the metabolites found to have strong correlations to phthalate levels in urine. Briefly, lists of metabolites with significant associations with phthalate urine biomarkers and prominent correlations (*r* > 0.3) were constructed and matched to their Human Metabolome Data Base (HMDB) identifiers using MetaboAnalyst (39). The online “Pathway Analysis” tool in MetaboAnalyst allows for direct input of compound names and generates the corresponding HMDB, PubChem, and KEGG identifiers. Use of HMDB identifiers greatly improved performance and reliability of the online tools by eliminating any confusion due to metabolite synonyms. We also manually searched for the HMDB identifier when it was not automatically assigned by MetaboAnalyst. These HMDB lists were submitted for pathway analysis, using “Pathway overrepresentation analysis” for IMPALA with the following parameters: “Homo Sapiens” pathway library, “Hypergeometric Test,” “Relative-betweeness Centrality,” and “Used all compounds in the selected pathways” as our options in MetaboAnalyst. Finally, within the HMDB entry for most metabolites, a KEGG link (38) provided detailed pathway diagrams.

## Results

### Demographic and exposure characteristics

Pregnant women in this study were young (25.6 ± 4.0 years old) and many were overweight or obese (mean BMI 26.4 ± 4.9 kg/m^2^) prior to pregnancy (Table 1). The distributions of average gravity-adjusted phthalate metabolite concentrations during pregnancy for the subset of the CHAMACOS mothers included in this study are similar to the levels observed in the full cohort (11, 12). Detection frequencies of phthalate metabolites during pregnancy were 90–100%. MEP had the highest urinary concentrations during pregnancy. Other common urine metabolites included MBP, MEHHP, and MECPP. The exposure in CHAMACOS pregnant women were comparable to trends seen in the general U.S. population (11).

**Table 1:** Characteristics and phthalate metabolite concentrations for CHAMACOS mothers (*N* = 115).

**Characteristics**	**Mean ±SD**	**Median**	**Min-Max**
Age, years	25.6 ± 4.0	25	18–37
BMI (kg/m2)	26.4 ± 4.9	25.1	17.7–45.5
**PHTHALATE METABOLITE CONCENTRATIONS (ng/mL)**			
**LMW**			
MEP	347.4 ± 623.0	161.1	0.9–5004
MBP	42.8 ± 69.2	25.9	1.8–597.1
MiBP	5.1 ± 6.1	3.0	0.1–37.4
LMW Sum	426.8 ± 690.5	209.6	13.3–5536.9
**DEHP**			
MEHP	6.5 ± 11.1	3.9	ND-94.2
MEHHP	31.1 ± 80.5	17.1	2–781.2
MEOHP	23.8 ± 60.4	13.8	0.2–588
MECPP	51.9 ± 120.5	27.4	5.8–1102.8
DEHP Sum	111.1 ± 264.4	63.3	9.1–2516.9
**HMW**			
MBzP	11.5 ± 13.0	7.2	ND-90.7
MCPP	2.4 ± 1.8	1.9	ND-9.6
MCOP	3.8 ± 2.9	3.2	ND-22.4
MCNP	2.3 ± 1.7	2.0	ND-10.2
HMW Sum	132.2 ± 266.6	83.1	14-2533.6

*All measures were specific gravity adjusted*.

*ND, Non-Detected; MEP, monoethyl phthalate; MBP, mono-n-butyl phthalate; MiBP, mono-isobutyl phthalate; LMW, low molecular weight metabolites; MEHP, mono(2-ethylhexyl) phthalate; MEHHP, mono(2-ethyl-5-hydroxyhexyl) phthalate; MEOHP, mono(2-ethyl-5-oxohexyl) phthalate; MECPP, mono(2- ethyl-5-carboxypentyl) phthalate; DEHP, di-2-ethylhexyl phthalate; MBzP, monobenzyl phthalate; MCPP, mono(3- carboxypropyl) phthalate; MCOP, monocarboxyoctyl phthalate; MCNP, monocarboxynonyl phthalate; HMW, high molecular weight metabolites; BMI, body mass index*.

### Urine and plasma metabolome

Among the 415 metabolic compounds targeted by our metabolomics platform, multiple lipid species were assessed including fatty acids, acylglycerophospholipids, neutral lipids, ether lipids, eicosanoids, sterols, and sphingolipids as well as polar metabolites that encompass major metabolic pathways such as glycolysis, TCA cycle, amino acid, pentose phosphate, hexosamine, polyamine, nucleotide, glycan, glucuronate, and inositol metabolism. We characterized 264 non-polar metabolites in plasma, as well as 151 polar metabolites in both plasma and urine samples. Detailed information on the assessed metabolites in plasma and urine is presented in Supplemental Table 1.

**Figure 1:**
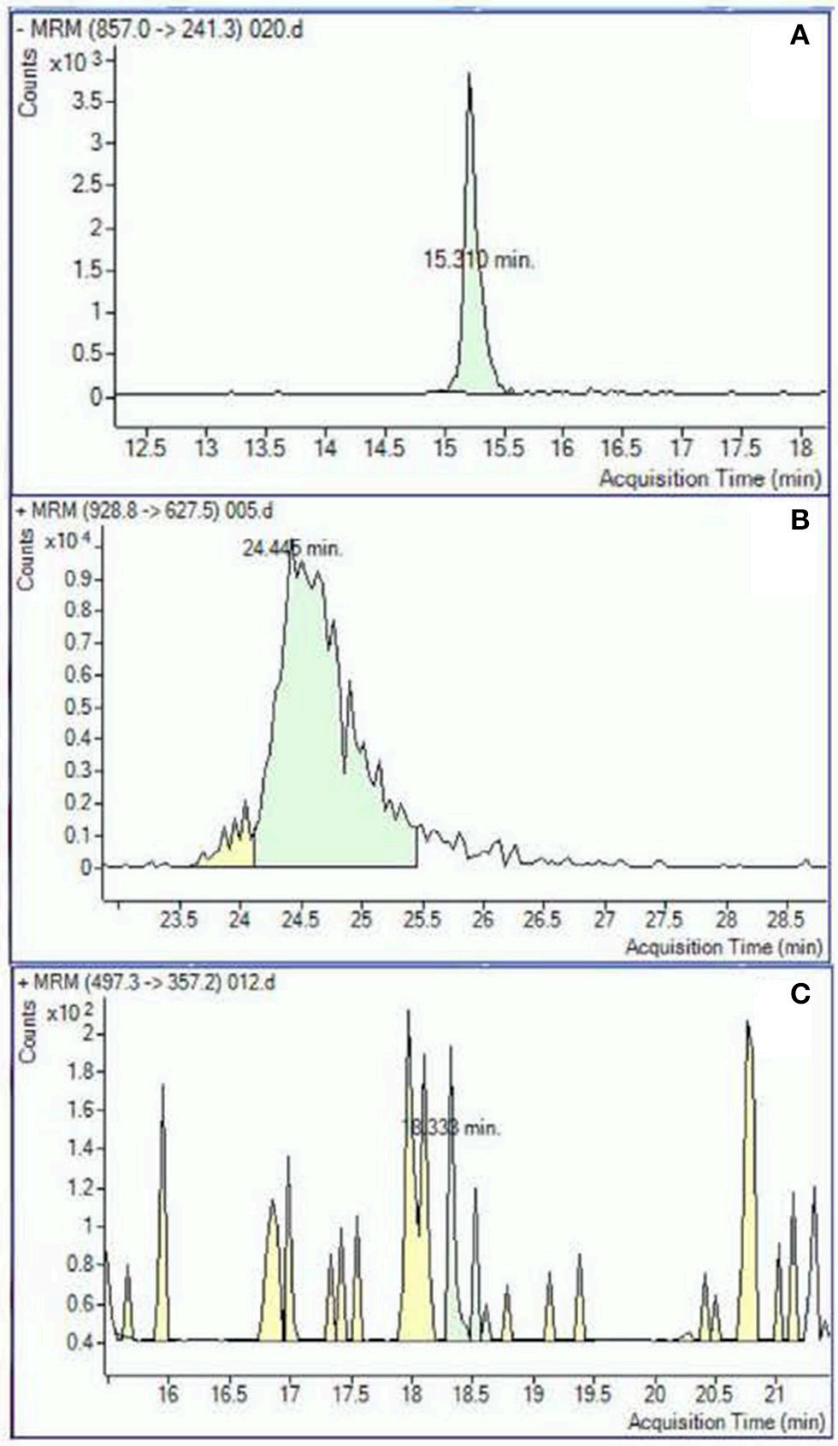
Examples of the metabolomic compounds considered to be acceptable for analysis that had peaks either clearly defined **(A)** or sufficiently defined **(B)**. Spectrograms similar to the example **(C)** were excluded.

**Figure 2:**
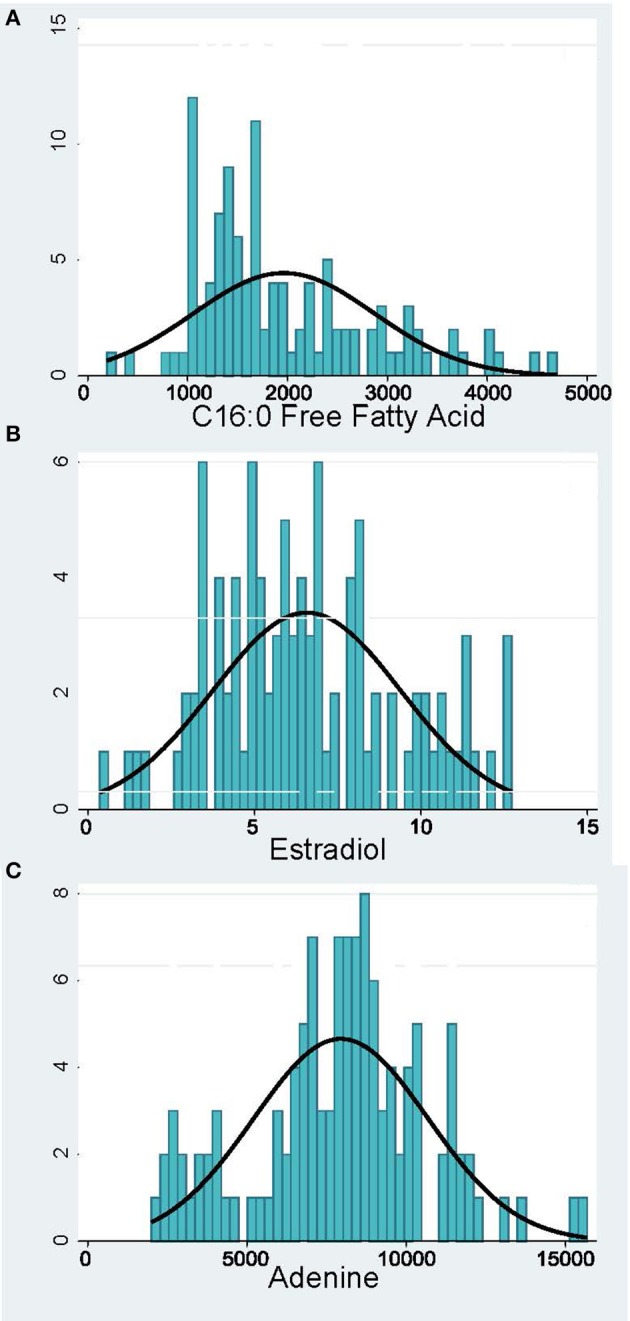
Distribution of representative metabolomic markers: **(A)** Inflammation and Lipid Biosynthesis, **(B)** Hormones, **(C)** Nucleotide Metabolism. The x-axis shows the relative abundance of the metabolite and the y-axis—the number of pregnant women.

Peak analysis was performed on high quality and consistent spectrographic peaks (Figure 1) which resulted in 138 plasma and 40 urine metabolites to be included for further analysis. Examples of distributions of several metabolic markers in plasma are shown in Figure 2. Free Fatty Acid (C16:0 FFA) is frequently related to inflammation and involved in lipid biosynthesis, high relative abundances of this metabolite were found in fewer than half of the samples (Figure 2A). For estradiol hormone, less abundance was observed overall but with a more normal distribution (Figure 2B). In contrast, adenine (reflecting nucleic acid degradation) was found in greater abundance across participant samples. We expected the relative abundance to be different in plasma and urine. Indeed, ribose 1,5-bisphosphate (R15BP) was the only metabolite that had the abundance appreciably correlated in both tissues (*r* = 0.33).

### Association of phthalate exposure and metabolic profiles in pregnant women

**Figure 3:**
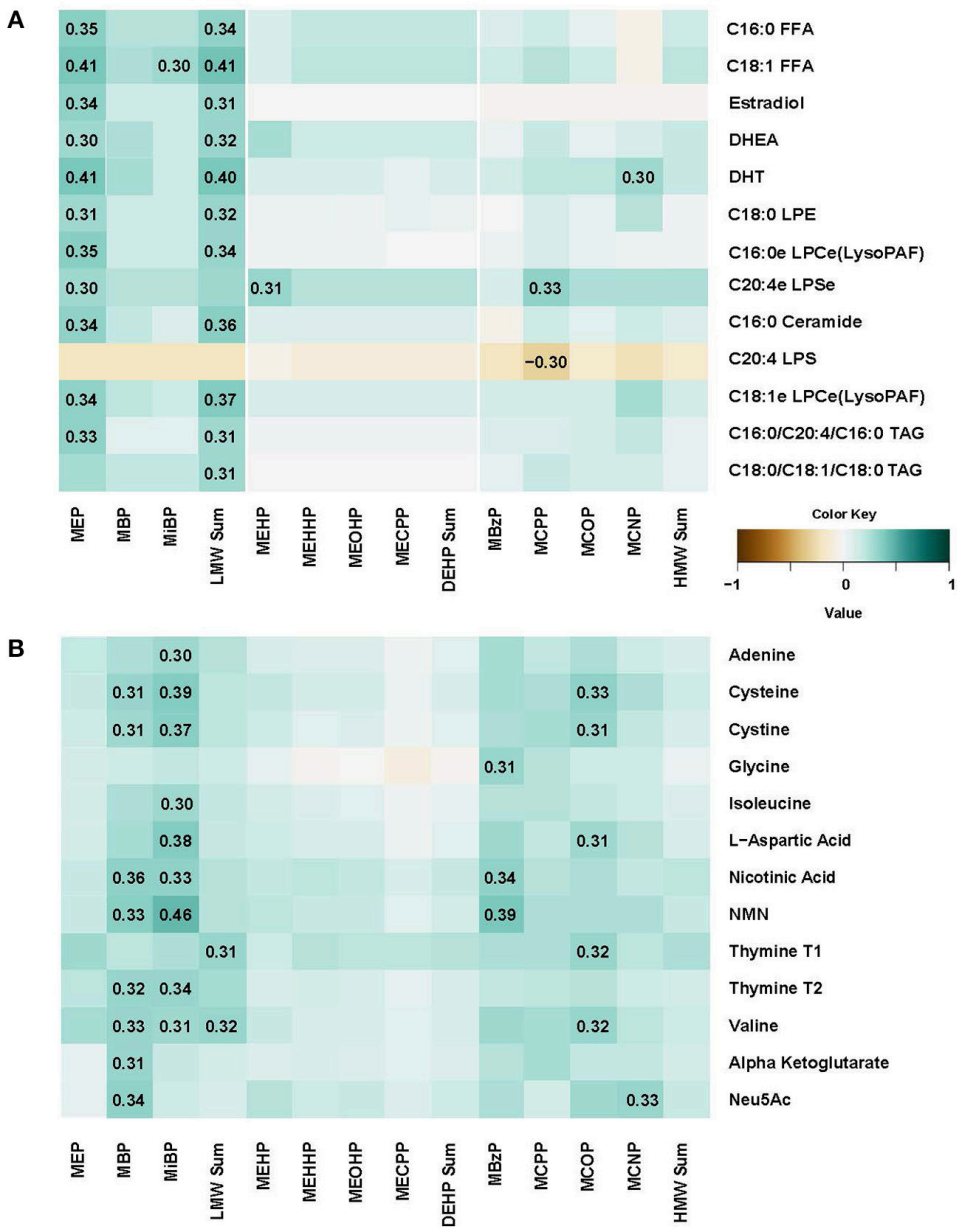
Spearman correlation matrix between concentrations of eleven urine phthalate metabolites and metabolomic markers in plasma **(A)** and urine **(B)**.

In plasma, several free fatty acids, hormones, lysolipids, ceramide and triacylglycerol metabolites were correlated with MEB and LMW Sum urine levels (Figure 3A). We also conducted regression analysis, and many metabolites (*N* = 72) were significantly associated with phthalates including 23.6% (*N* = 17) with MEP, confirming observations with correlation analysis (Supplemental Table 2). Most of the associations were positive, with *p*-values ranging from 0.0002 to 0.049, and for 15 metabolites that were negatively associated, *p*-values ranged from −0.0003 to −0.048. All positive and negative betas were relatively small (−0.321 to 0.478). Further, we conducted sensitivity analysis adjusting for maternal BMI and parity, two variables that were previously identified as the most relevant to phthalate metabolite levels (11). The results were almost identical to non-adjusted regression model. However, when we did adjustment for multiple comparisons (FDR) significant findings were attenuated.

In urine, N-acetylneuraminic acid (Neu5AC), amino acids, nicotinic acid metabolites, adenine and thymine were correlated with many phthalate metabolite concentrations including MBP, MiBP, MBzP, and MCOP (Figure 3B). Regression analysis confirmed that there were 171 significant associations, with multiples for different phthalate urine biomarkers (Supplemental Table 3). Moreover, even after FDR correction, 9 positive associations remain significant (*q*-values ranging from 0.0001 and 0.0451) in the model adjusted for BMI and parity. For instance, nicotinamide mononucleotide (NMN) was associated with MiBP (beta = 0.452), MBP (beta = 0.433), MBzP (beta = 0.380), MCPP (beta = 0.472), and MCNP (beta = 0.481). Cystine and cysteineT2 were associated with MiBP (betas 0.282 and 0.319, respectively) and cysteine was also associated with MCPP (beta = 0.341). L-Aspartic acid association with MiBP also remained significant after FDR adjustment (beta = 0.338, *q* = 0.0195).

In the PCA analysis, 16 of the 40 PCs explained 95% of the variance, and were included in the regression analysis with phthalate metabolite concentrations. After FDR adjustment, only PC1 that explained ~50% of the variance was significantly associated with urinary phthalate metabolites (MBzP in the crude and adjusted for BMI and parity models and MCPP in the crude model). There was no clear clustering by metabolite (values of the PC loadings ranged from −0.24 to −0.05).

### Metabolomic markers and obesity in CHAMACOS women

Women in the CHAMACOS cohort had a high prevalence of obesity before pregnancy (>50% were overweight or obese), with their BMI ranging broadly from 17.7 to 45.5 (Table 1). We explored the relationship of the pre-pregnancy BMI with targeted metabolomics markers characterized in this study to establish their biological relevance. A complete list of the associations is presented in Supplemental Table 4. Most of the coefficients for metabolomic compounds in plasma, among 25 that were statistically significant, were negative. These compounds included glycine (beta = −1.92; *p* = 0.0004); acetylcarnitine (beta = −1.69; *p* = 0.002); isoleucine (beta = −1.93; *p* = 0.01); cholesterol (beta = −3.04; *p* = 0.01); tryptophan (beta = −3.24; *p* = 0.02). Mevalonic acid had a strong positive association with BMI (beta = 9.58.24; *p* = 0.009). In contrast, nicotinic acid was the only urine metabolomic compound that has shown a statistically significant association with BMI (beta = 4.01, *p* = 0.002).

### Metabolic pathways associated with phthalate exposure

**Figure 4:**
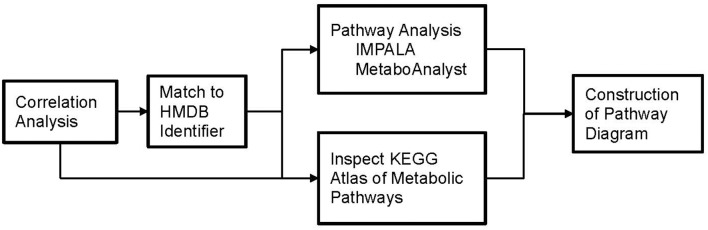
Flowchart depicting our approach to identify which metabolic pathways are influenced by phthalate exposure in pregnant women.

**Figure 5:**
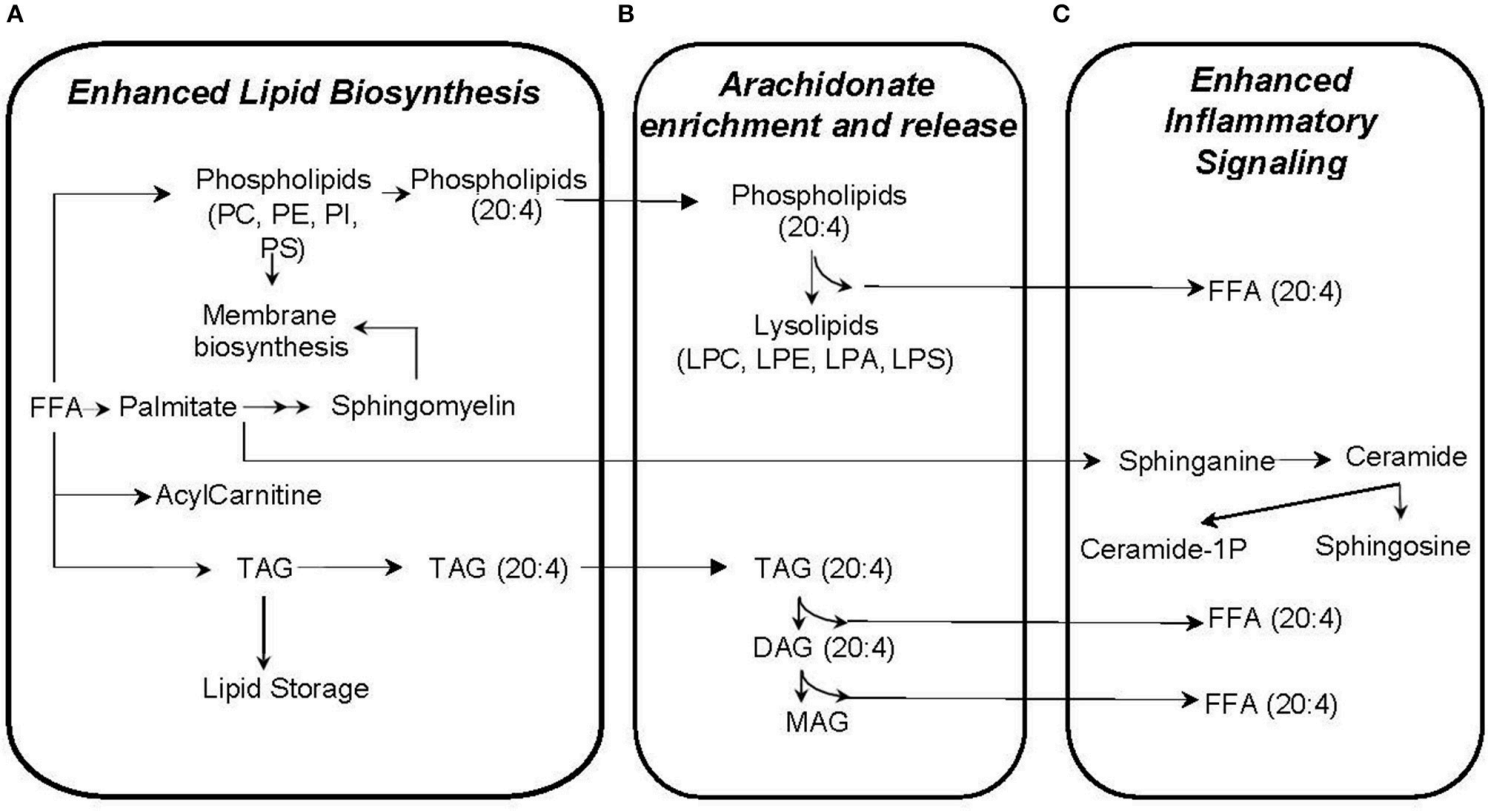
Plasma metabolites correlated with phthalate exposure likely reflect enhanced lipid biosynthesis **(A)**, arachidonate enrichment and release **(B)**, and inflammatory signaling **(C)**.

Our approach to identifying which metabolic pathways are influenced by phthalate exposure is depicted in Figure 4. As shown in Figure 5, lipid biosynthetic and degradation products and arachidonate containing lipids were correlated with phthalate exposure markers (urine phthalate metabolite concentrations). Metabolites reflecting components of the biological membrane, phosphatidylcholine (PC), phosphatidylethanolamine (PE), phosphatidylserine (PS), and phosphatidylinositol (PI), cholesterol and sphingomyelin were also correlated with phthalate exposure, most notably MEP, MBP, MiBP, and LMW Sum levels in urine (Figure 5A). Multiple triacylglyceride (TAG) species were associated with many phthalate metabolites measured in urine (Figure 5A). TAGs serve as a reservoir for energy storage in the form of esterified fatty acids, existing as lipid droplets found abundantly in adipose tissue. Palmitoyl carnitine (16:0 AC) and steroyl carnitine (18:0 AC), conjugated forms of FFAs involved in beta-oxidation, also correlate with previously measured phthalate urine metabolite concentrations (Figure 5A). Metabolic products formed from phospholipid and TAG phospholipase mediated degradation, including lysophosphatylcholine (LPC), lysophosphatidylethanolamine (LPE), lysophosphatidate (LPA), lysophosphatidylserine (LPS), diacylglycerides (DAGs), monoacylglycerides (MAGs), and FAA were associated with phthalate urine levels (Figure 5B). Ceramide, ceramide-1phosphate, sphingosine and sphingosine 1-phosphate, metabolites that are intermediates of sphingosine biosynthesis and members of an inflammatory signaling pathway, also appear to be associated with the phthalate exposure (Figure 5C). The same was observed for a subset of phospholipids, PI and PS and TAGs containing arachidonate (20:4). Similarly, arachidonate, the free fatty acid (FFA) precursor for eicosanoid biosynthesis also demonstrated noticeable relationship with phthalate exposure markers including MEP, MBP, MiBP, LMW Sum, MEHP, MEHHP, MECHP, MECPP, DEHP Sum, and HMW Sum (Figure 5C).

### Plasma hormone metabolites

**Figure 6:**
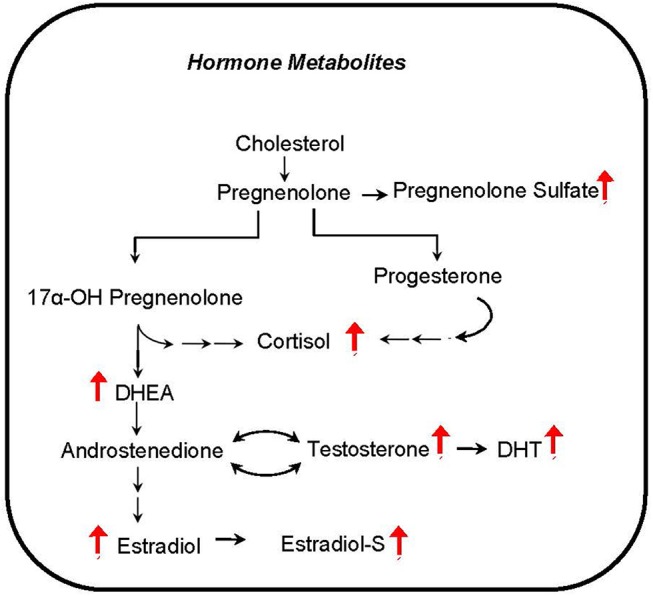
Hormone metabolites correlate with phthalate exposure. Red arrows indicate upregulation of corresponding metabolite.

Several hormones that are known to influence metabolism correlate with phthalate exposure markers in CHAMACOS pregnant women. The androgen metabolites dihydrotestosterone (DHT) and dehydroepiandrosterone (DHEA) were associated with many of the detected phthalate metabolites (Figure 6). In addition, MEP and the sum of LMW phthalate metabolite concentrations appear to be aligned with many steroid hormones including cortisol, a stress hormone, estradiol and testosterone, hormones that regulate sex development, and pregnenolone- and estradiol-sulfate hormones.

### Plasma and urinary nucleic acid degradation products

**Figure 7:**
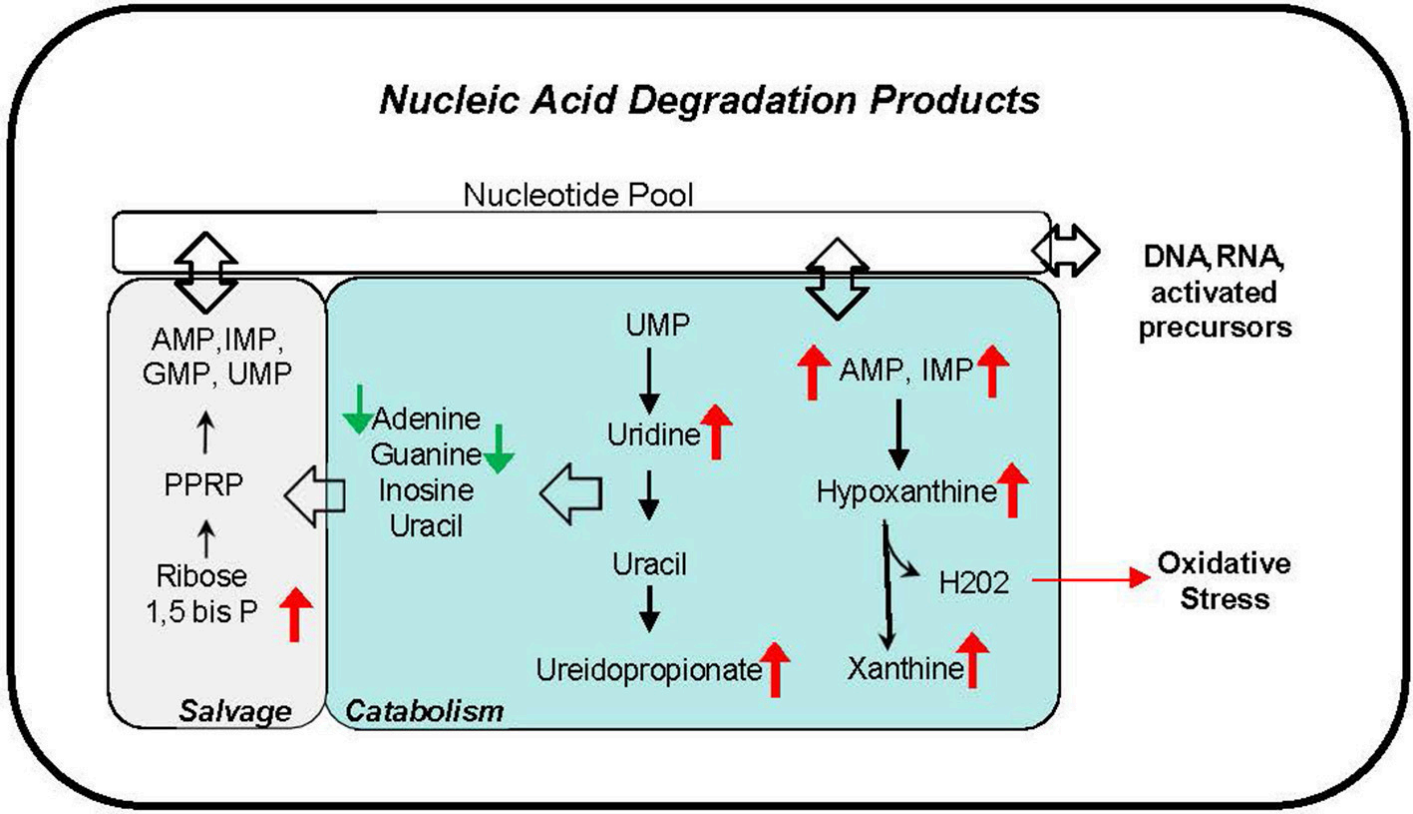
Nucleic acid degradation products correlate with phthalate exposure in plasma and urine. Red arrows indicate upregulation, and green arrows indicate downregulation of corresponding metabolite.

Phthalate exposure markers as measured by concentration of 11 urine metabolites were associated with several nucleic acid degradation products including pyrimidine metabolites, thymine, uridine and ureidopropionate, and purine metabolites AMP, IMP, hypoxanthine, and xanthine (Figure 7). The correlations ranging *r* = 0.31–0.46 are observed with most phthalate urine levels including MBP, MiBP, MEHP, MEHHP, MEOHP, MECPP, DEHP Sum, MBzP, MCPP, MCOP, and HMW Sum. The accumulation of nucleic acid degradation products suggests an increased nucleic acid turnover and/or impaired salvage with phthalate exposure. Oxidative stress may also be exacerbated with phthalate exposure. Hydrogen peroxide is concomitantly generated with the conversion of hypoxanthine to xanthine by xanthine oxidase.

## Discussion

We determined metabolomic profiles in plasma and urine from pregnant women and examined their association with phthalate urine biomarkers during gestation and pre-pregnancy obesity. We observed mostly positive associations with higher phthalate concentrations which could be suggestive of increased inflammation, changes in lipid biosynthesis as well as hormone and nucleic acid metabolism with higher urine phthalate levels, providing additional insight into the underpinnings of how phthalates may contribute to obesity and other health outcomes (40). We also demonstrated significant negative relationship of over 20 of metabolomics compounds in plasma with maternal pre-pregnancy BMI. This finding is consistent with other studies that reported that women's obesity before pregnancy is related to their metabolic status (22).

In our study, women with higher phthalate exposure tended to have enriched arachidonate content in lipids (Figure 5), a change that perhaps enhances the inflammatory response in much the same way a high n-6:n-3 fatty acid ratio from a Western diet contributes to inflammation (41). Arachidonate is the precursor for eicosanoid biosynthesis (42), and release of arachidonate from complex lipids is achieved through the action of phospholipases which generate lipid degradation products, the same ones which also correlate with phthalate exposure. In animal models, the association of phthalate biomarkers with increased inflammatory response has been clearly demonstrated with detrimental effects on the neonate (43–45). For example, as with our study, rats exposed to phthalate also have increased arachidonate containing phosphatidylcholine (46). Multiple studies have evaluated phthalate exposure in pregnant women (47–49), and several have also demonstrated relationships with oxidative stress markers across pregnancy (11, 50). Our results suggest that enhanced inflammatory response from phthalate exposure is linked to a biological feature further upstream, namely the arachidonate availability, storage and release to propagate eicosanoid biosynthesis. These cellular events are supported by the correlation of several lipid degradation products, DAGs, MAGs, lysolipids, and arachidonate (Figure 5B).

Most lipid metabolites that correlate with phthalate exposure have a positive association suggesting that phthalates may have a mechanistic relationship with the up-regulation of lipid synthesis pathways or down-regulation of lipid degradation pathways. In contrast, one of the negatively associated lipid metabolites, 20:4 LPS (Figure 3A), a degradation product of phosphatidylserine may indicate a compensatory role with phthalate exposure, regulating lipid redistribution and inflammation similar to shown in animal studies (51, 52).

Our results also recapitulate a mechanistic model which may explain how phthalate exposure induces lipid biosynthesis and weight gain. The correlation of biosynthetic lipids with phthalate exposure is consistent with previous findings (53–55). For example, recent experiments identified multiple genes associated with lipid metabolism that are up-regulated with phthalate exposure in rat embryo and human fetal gonad cultures, such as liver-X-receptor alpha (*LXR*α), sterol regulatory element-binding protein *(SREBP) 1c*, and *SREBP2* (53-55). Muczynski et al., showed that phthalates increase the levels of mRNA for *LXR*α, and *SREBP* members, possibly up-regulating lipid and cholesterol synthesis in human fetal gonads (53). Consistent with the up-regulated genes, phospholipids and neutral lipids are increased in rats with phthalate exposure (46). Our results showing correlations between higher phthalate exposure with products of lipid biosynthesis provides evidence that phthalate-induced disruption of upstream transcription factors (LXR, SREBP1c, SREBP2) may also occur in humans, contributing to increased lipid biosynthesis and obesity. In addition, several correlating sphingolipids such as ceramide, ceramide-1-phosphate, sphingosine, and sphingosine-1-phosphate may serve as signaling molecules that influence obesity, diabetes, metabolic syndrome (56–58), oxidative stress and inflammatory signaling (59). Previous reports describe the effects of phthalates on sphingolipid metabolism in rat brain linked with hippocampal development (60) and liver toxicity (61), but additional research is required to understand their role in maternal-neonate health and obesity.

Characteristics of four metabolomic compounds (cysteine T2, cystine, NMN and L-aspartic acid) that showed strong associations with phthalate metabolite concentrations in urine in our study (they remained statistically significant after multiple comparison adjustment), also support the involvement with these metabolic pathways. For instance, cysteine has many biological functions, including antioxidation (62). Aside from its oxidation to cystine (oxidized dimeric form of cysteine), cysteine participates in numerous posttranslational modifications. The nucleophilic sulfhydryl group allows cysteine to conjugate to other groups. Cystine is found in high concentrations in digestive enzymes and in the cells of the immune system and connective tissues. Cystinuria is an inherited disorder of the transport of cystine resulting in an excess of cysteine in the urine and the formation of cystine stones (63). Nicotinamide Mononucleotide, NMN, a key NAD^+^Intermediate was shown to be effective in treating diet and age-induced diabetes in mice (64). NMN also enhances hepatic insulin sensitivity and restores gene expression related to oxidative stress, inflammatory response, and circadian rhythm. L-aspartic acid is an α-amino acid that is used in the biosynthesis of proteins, and participates in the urea cycle (65).

Our observations of metabolomic profiles in pregnant women are consistent with findings by Zhang et al. in phthalate exposed men (21). First, while prostaglandin metabolites were increased in these male subjects, we observed that upstream metabolites to eicosanoid biosynthesis were correlated with phthalate exposure- namely arachidonate and arachodonate-containing lipids in plasma of pregnant women in our study. Second, the associations we observed between phthalate exposure biomarkers and acylcarnitines is consistent with increases in male urine and female plasma with higher phthalate exposure, suggesting that mitochondrial function may be influenced by phthalate exposure. Further experiments are necessary to determine whether acylcarnitines are increased because beta-oxidation is up-regulated as concluded by Zhang et al., or if it may be a result of mitochondrial dysfunction (66). Third, urinary Neu5AC and N-acetylmannosamine may indicate protein glycosylation impairment (67, 68) causing the aminoglycans to accumulate in urine with phthalate exposure. Last, several urinary amino acids were elevated in men and women with higher phthalate exposure, including cystine (Figure 3B), alanine and tryptophan (data not shown), a potential sign of disrupted amino acid metabolism.

Another intriguing finding from our analysis is the prominent positive correlation of steroid hormones and sulfated hormones with phthalate exposure biomarkers. Steroid signaling is considered to be critical for organizing embryonic development (69). Furthermore, maternal steroid sulfonation is a common metabolic pathway during embryonic development in many vertebrates (70). Our results suggest that phthalates have the potential to disrupt the close hormonal communication between the developing placenta and fetus. Phthalates are suspected endocrine disruptors, but their precise relationship with sex steroid hormone levels is not sufficiently understood. Although genes related to steroid metabolism are up-regulated in phthalate exposed rats (54), prenatal exposure is associated with lower circulating testosterone and aldosterone levels in adult male offspring and estradiol in the female (71). Additionally, as it was shown in zebrafish and in human adrenal cell line, testosterone may be decreased with phthalate exposure (72). In contrast, our results show that pregnant women with higher levels of phthalates tend to have increased steroid hormone levels. It may be possible that these hormone levels are related to cholesterol availability which is regulated by *SREBP1c* and activated by phthalate exposure (55). The discrepancy in hormone level changes in previous reports and our findings may reflect experimental differences in animal or cell culture models compared to human subjects, or perhaps related to dose effects (46).

Our pathway analysis also suggests a novel relationship with nucleic acid metabolism, an association in line with our previous report linking phthalate exposure and differential DNA methylation in the same cohort (12, 73, 74). The correlation of several nucleotides and nucleosides in plasma and urine with higher exposures suggests that phthalates may influence degradation, salvage, and elimination pathways of nucleic acid metabolism. Purines, hypoxanthine and xanthine data suggest activation of purine degradation by xanthine oxidase which also produces hydrogen peroxide, a potential source of oxidative stress (75–78). Additional research is needed to establish whether the alterations of nucleic acid metabolites reflect enhanced DNA repair mechanism to combat phthalate induced injury (79).

Our study has several strengths and some limitations. It was conducted in a relatively homogeneous Mexican-American cohort of pregnant women, potentially reducing the impact of unaccounted confounding (80). The women had levels of phthalate exposure typical for many American cohorts; thus, making the findings regarding their metabolomic profile fairly generalizable. While this study is comparable in size with other published metabolomics studies, confirmation in larger diverse cohorts will be required in the future. We employed a targeted metabolomics analysis with more than a hundred measured metabolites across two sample types which provided an opportunity to ascertain the relationship between phthalate urine metabolite concentrations with the urine and plasma metabolomes. Our study of pregnant women is only the first step to explore the environmental metabolomics in this cohort with extensive exposure data for pesticides, flame retardants and other chemicals measured during pregnancy and in their children, whom we have followed through age 18 years to date. In the future, pending additional funding, we plan to expand the number of participants, characterize their metabolome at multiple time-points and explore the relationship of identified metabolomics markers with health outcomes such as obesity, hormonal, and reproductive outcomes in children that may be affected by early life phthalate exposure.

In conclusion, this study for the first time characterized the metabolome of urine and plasma in pregnant women and explored their relationships with urine phthalate concentrations measured by standard methodologies as a biomarker of phthalate exposure. We identified new candidate metabolomic markers associated with phthalates that in the future can be explored in relation to various health outcomes. Pathway analysis indicates potential alterations in lipid biogenesis, inflammation, sphingolipid signaling and nucleotide degradation which could exacerbate oxidative stress. These results provide new insights into the relationship between phthalate exposure and metabolomic profiles during gestation.

## Author contributions

KH advised MZ in the initial statistical analyses and planning of the experiments. MZ, BF, and VT prepared samples, conducted experiments, and contributed to the data analysis. NH designed the study and worked on the paper with MZ and GT. DL conducted pathway analysis and helped with the writing of the manuscript. RG and GT were involved in the statistical analyses. BE, AB, DN contributed to the data interpretation and preparation of the manuscript.

### Conflict of interest statement

The authors declare that the research was conducted in the absence of any commercial or financial relationships that could be construed as a potential conflict of interest. The handling editor declared a past co-authorship with one of the authors NH.
