# Supplementary material for: Metabolomic Markers of Phthalate Exposure in Plasma and Urine of Pregnant Women
Source: Front Public Health. 2018 Oct 22;6:298. doi: 10.3389/fpubh.2018.00298 (PMC6204535; doi:10.3389/fpubh.2018.00298)
Supplement: Supplementary file 1 [file Table_1.docx]

| **Supplemental Table 1.** Distribution of metabolomic markers analyzed with selected  reaction monitoring liquid chromatography and triple quadrupole mass spectrometry in pregnant women (n=115). Plasma Nonpolar Positive metabolites were normalized to C12 MAGE, Plasma Nonpolar Negative metabolites to PDA, and Plasma Polar and all Urine data were normalized to D3 N15 Serine | | | | | |
| --- | --- | --- | --- | --- | --- |
| **Metabolite** | **Mean** | **Standard Deviation** | **Median** | **Min** | **Max** |
| **Urine - Polar Positive Metabolites** | | | | | |
| 3-Ureidoproprionate | 97,219.0 | 11,488.5 | 50,190.5 | 705.2 | 580,801.5 |
| Adenine | 43,287.6 | 7,946.7 | 23,747.2 | 5,043.2 | 860,451.5 |
| Adenosine | 8,877.9 | 1,131.6 | 5,294.1 | 13.9 | 93,392.9 |
| Asparagine | 95,802.4 | 9,056.4 | 58,981.5 | 7,072.9 | 551,538.4 |
| Creatine | 119,122.2 | 13,682.0 | 59,578.7 | 1,969.5 | 677,502.3 |
| Cysteine T2 | 43,536.5 | 7,169.5 | 24,925.1 | 2,579.9 | 601,194.4 |
| Cystine | 147,778.9 | 20,985.4 | 84,364.4 | 12,281.3 | 2,194,997.1 |
| Cytosine | 42,916.2 | 4,074.7 | 27,290.2 | 5,614.6 | 316,577.5 |
| Fumarate | 11,417.7 | 3,546.3 | 4,645.2 | 769.7 | 405,708.8 |
| Glutamine | 1,893,704.7 | 197,884.7 | 1,238,861.5 | 42,839.8 | 14,043,962 |
| Glycine T1 | 522,330.1 | 57,841.7 | 353,635.3 | 201.8 | 3,758,670.3 |
| Histidine | 1,781,142.0 | 179,859.8 | 1,118,753.0 | 86,359.4 | 10,508,088 |
| Isoleucine | 11,606.9 | 1,186.0 | 7,000.0 | 474.7 | 63,901.1 |
| L-Alanine | 1,190,796.4 | 127,748.7 | 632,355.2 | 17,578.1 | 6,571,571.1 |
| L-Aspartic Acid | 150,910.3 | 17,684.9 | 86,189.9 | 6,325.0 | 1,336,377.4 |
| Nicotinic Acid | 14,706.3 | 4,197.3 | 5,960.3 | 1,382.4 | 467,700.5 |
| Nicotinamide Mononucleotide | 7,253.6 | 1,170.7 | 3,463.7 | 73.3 | 117,604.8 |
| Phenylalanine | 74,871.2 | 8,249.2 | 45,058.2 | 9,146.8 | 647,717.8 |
| Thymine T1 | 4,733.9 | 1,738.7 | 1,922.6 | 551.7 | 199,561.7 |
| Thymine T2 | 46,147.8 | 11,341.4 | 23,569.8 | 4,337.9 | 1,285,466.5 |
| Tryptophan | 124,998.0 | 35,931.1 | 49,931.8 | 8,649.9 | 4,049,555.9 |
| Uracil | 6,873.1 | 2,587.1 | 2,531.0 | 492.4 | 290,906.1 |
| Valine | 355,565.7 | 32,499.1 | 244,812.8 | 25,257.5 | 1,733,194.2 |
| **Urine - Polar Negative Metabolites** | | | | | |
| Alpha Ketoglutarate | 935,114.9 | 174,373.3 | 394,799.7 | 34,086.9 | 14,994,340 |
| Citrate | 481,458.4 | 81,751.2 | 130,240.6 | 634.9 | 4,475,506.2 |
| Cytidine Monophosphate | 11,112.9 | 2,907.1 | 3,259.8 | 0.0 | 274,188.9 |
| Glucose New | 237,248.4 | 85,029.3 | 36,439.3 | 530.6 | 8,703,679.1 |
| Glucose Old | 684,942.1 | 149,540.8 | 219,549.3 | 1,190.5 | 13,362,643 |
| Glucuronic Acid | 541,848.7 | 92,498.0 | 162,362.8 | 0.0 | 5,183,131.6 |
| Hypoxanthine | 509,399.0 | 85,376.8 | 172,895.4 | 619.9 | 6,401,774.8 |
| Lactic Acid | 364,218.6 | 134,717.7 | 62,306.0 | 3,690.5 | 12,582,733 |
| N-Acetyl mannosamine | 101,164.8 | 26,702.7 | 28,523.2 | 238.2 | 2,125,691.1 |
| N-Acetyl neuraminic acid | 64,128.6 | 15,300.7 | 15,893.1 | 258.4 | 1,104,595.1 |

| Pantothenate | 414,641.7 | 80,333.5 | 131,595.1 | 11,021.7 | 5,052,769.2 |
| --- | --- | --- | --- | --- | --- |
| Phosphoenolpyruvic Acid | 20,144.7 | 3,252.6 | 8,853.8 | 229.9 | 230,836 |
| Ribose-15- Biphosphate | 1,190,312.0 | 352,405.2 | 234,810.2 | 19,314.2 | 29,177,258 |
| Thymine | 26,401,794.2 | 4,532,764.0 | 11,939,352.0 | 240,595.2 | 335,500,000 |
| Uracil | 225,695.4 | 60,027.1 | 41,362.4 | 992.1 | 4,319,480.6 |
| Uridine | 2,388,707.1 | 444,520.6 | 832,541.1 | 121,757.7 | 32,681,946 |
| Xanthine | 366,305.7 | 96,405.5 | 137,397.4 | 2,142.9 | 9,543,754.6 |
| **Plasma - All Polar Metabolites** | | | | | |
| Acetyl CoA 13C | 1,550.7 | 63.2 | 1,508.4 | 0.0 | 4,035.0 |
| Adenosine Monophosphate | 6,648.5 | 393.4 | 5,892.3 | 1,092.6 | 19,621.2 |
| Cytidine Triphosphate | 5,448.9 | 271.8 | 5,868.1 | 390.1 | 12,572.2 |
| Deoxyuridine Triphosphate | 990.0 | 36.3 | 925.8 | 324.1 | 2,576.1 |
| Hypoxanthine | 3,316.9 | 366.4 | 758.2 | 57.2 | 11,568.8 |
| Inosine | 1,338.1 | 77.1 | 1,083.5 | 116.3 | 3,908.1 |
| Lactic Acid | 36,203.5 | 3,562.5 | 19,773.4 | 1,110.5 | 154,767.6 |
| N-  (phosphonomethyl) glycine 1 | 40,133.6 | 3,474.0 | 28,054.2 | 2,142.9 | 125,864.2 |
| N-  (phosphonomethyl) glycine 2 | 2,067.6 | 177.8 | 1,815.8 | 34.4 | 6,478.3 |
| Ribose-15- Biphosphate | 103,902.6 | 9,065.1 | 73,664.2 | 19,705.8 | 692,760.9 |
| Thymine | 5,912.6 | 445.0 | 5,160.5 | 226.6 | 23,063.4 |
| Uridine | 2,522.2 | 240.7 | 1,334.5 | 113.4 | 9,567.1 |
| Xanthine | 2,249.9 | 347.6 | 1,330.3 | 85.2 | 34,974.9 |
| Glycine T2 | 766.8 | 136.8 | 381.9 | 3.9 | 12,693.7 |
| Glycine T1 | 4,106.2 | 799.7 | 1,112.1 | 5.7 | 66,982.9 |
| L-Alanine | 55,000.4 | 3,165.3 | 58,523.9 | 59.6 | 151,356.7 |
| Serine | 31,549.1 | 2,337.8 | 32,488.3 | 219.2 | 155,109.1 |
| Proline | 599,514.8 | 38,087.2 | 566,666.7 | 4,673.6 | 2,148,618.9 |
| Valine | 76,320.8 | 5,520.3 | 66,723.4 | 1,201.5 | 281,936.7 |
| Cysteine T2 | 2,194.6 | 138.2 | 2,093.4 | 20.6 | 5,917.2 |
| Creatine | 4,834.7 | 316.1 | 4,514.5 | 111.9 | 27,031.2 |
| Isoleucine | 7,826.8 | 552.6 | 7,784.9 | 77.9 | 38,121.4 |
| 3-Ureidoproprionate | 60.9 | 3.6 | 52.7 | 6.0 | 191.6 |
| Asparagine | 323.0 | 18.6 | 299.3 | 39.0 | 1,001.9 |
| L-Aspartic Acid | 16,094.7 | 1,952.0 | 10,354.6 | 16.8 | 99,556.1 |
| Adenine | 7,953.6 | 263.5 | 8,093.5 | 1,999.7 | 15,674.2 |
| Glutamine | 70,157.7 | 3,310.1 | 73,917.1 | 5,847.5 | 185,279.9 |
| Lysine | 6,457.6 | 964.8 | 3,524.7 | 649.5 | 69,708.7 |

| Glutamic Acid | 44,352.1 | 5,460.2 | 35,254.3 | 13.1 | 468,319.5 |
| --- | --- | --- | --- | --- | --- |
| Mevalonic Acid | 534,662.4 | 18,603.2 | 474,492.5 | 353,541.7 | 1,225,814.5 |
| Guanine | 2,417.6 | 194.9 | 1,792.0 | 808.6 | 15,455.6 |
| Histidine | 87,529.2 | 5,129.3 | 90,862.0 | 1,015.7 | 305,609.0 |
| Phenylalanine | 52,880.1 | 3,913.9 | 52,935.2 | 1,040.1 | 310,285.8 |
| Arginine | 82,117.2 | 5,017.5 | 85,539.3 | 1,067.3 | 325,496.3 |
| Acetylcarnitine | 22,202.1 | 2,273.3 | 11,986.2 | 9.9 | 114,336.9 |
| tryptophan | 32,297.7 | 1,764.2 | 31,426.6 | 2,500.3 | 135,421.1 |
| NADP | 782.7 | 25.3 | 753.4 | 167.0 | 1,462.4 |
| NADPH | 965.3 | 32.3 | 967.8 | 138.5 | 2,245.5 |
| Acetyl-CoA T1 | 2,868.8 | 64.9 | 2,923.9 | 247.5 | 4,419.5 |
| Acetyl-Coa T2 | 4,291,967.3 | 104,425.9 | 4,468,425.5 | 300,431.1 | 6,870,007.2 |
| b-Hydroxyl-b- methylglutaryl-CoA | 203,781.9 | 3,863.3 | 201,147.1 | 47,960.6 | 360,702.6 |
| **Plasma - Nonpolar Negative Metabolites** | | | | | |
| C18:0/C16:0  ceramide-1- phosphate | 9.5 | 0.6 | 8.5 | 0.1 | 68.6 |
| C18:1/C16:0  ceramide-1- phosphate | 97.6 | 3.0 | 96.2 | 0.0 | 204.0 |
| C16:0 alkyl LPA | 1.6 | 1.0 | 0.4 | 0.0 | 101.2 |
| C16:0 FFA | 2,378.7 | 490.5 | 1,664.1 | 191.8 | 53,085.1 |
| C16:0 LPA | 55.0 | 2.5 | 52.4 | 0.0 | 181.8 |
| C16:0/C16:0 PI | 3.0 | 0.4 | 1.9 | 0.0 | 37.0 |
| C16:0/C18:1 PA | 13.1 | 0.5 | 11.9 | 0.6 | 41.8 |
| C16:0/C18:1 PI | 16.0 | 1.5 | 12.3 | 0.4 | 127.7 |
| C16:0/C20:4 PI | 9.2 | 0.7 | 7.6 | 0.3 | 53.1 |
| C18:0 FFA | 1,127.4 | 214.0 | 817.8 | 305.1 | 23,311.0 |
| C18:0 LPA | 12.0 | 0.8 | 10.2 | 0.1 | 53.3 |
| C18:0/C18:1 alkyl PA | 4.3 | 0.1 | 4.1 | 0.1 | 10.2 |
| C18:0/C18:1 alkyl PI | 0.7 | 0.1 | 0.5 | 0.1 | 4.4 |
| C18:0/C18:1 PA | 39.2 | 1.2 | 36.6 | 1.0 | 80.1 |
| C18:0/C18:1 PI | 20.0 | 1.6 | 16.6 | 0.9 | 113.0 |
| C18:0/C20:4 alkyl PA | 1.7 | 0.8 | 0.9 | 0.0 | 84.1 |
| C18:0/C20:4 alkyl PI | 0.7 | 0.1 | 0.5 | 0.0 | 5.2 |
| C18:0/C20:4 PA | 3.6 | 0.1 | 3.4 | 0.0 | 9.9 |
| C18:0/C20:4 PI | 41.7 | 2.9 | 35.9 | 2.4 | 182.5 |
| C18:1 alkyl LPA | 0.7 | 0.1 | 0.6 | 0.0 | 3.5 |
| C18:1 alkyl LPI | 1.8 | 0.2 | 1.2 | 0.0 | 11.2 |
| C18:1 FFA | 1,688.2 | 150.0 | 1,231.4 | 97.7 | 11,808.5 |
| C18:1 LPI | 0.3 | 0.1 | 0.1 | 0.0 | 11.6 |

| C20:4 FFA | 3.9 | 0.7 | 3.0 | 0.2 | 79.2 |
| --- | --- | --- | --- | --- | --- |
| C20:4 LPA | 2.7 | 0.3 | 2.5 | 0.0 | 29.0 |
| Cardiolipin C18:1/18:1/18:1/18:1 T2 | 1.3 | 0.1 | 1.2 | 0.0 | 7.2 |
| DHA | 548.5 | 160.3 | 92.9 | 17.9 | 16,665.4 |
| Phytanic Acid | 7,233.5 | 2,434.1 | 574.3 | 86.7 | 254,871.3 |
| **Plasma - Nonpolar Positive Metabolites** | | | | | |
| Estradiol | 6.6 | 0.3 | 6.3 | 0.3 | 15.1 |
| Testosterone Transition 1 | 2.1 | 0.4 | 0.1 | 0.0 | 13.1 |
| Testosterone Transition 2 | 1.2 | 0.0 | 1.2 | 0.1 | 2.6 |
| DHEA Transition 2 | 1.7 | 0.1 | 1.8 | 0.1 | 5.3 |
| DHEA Transition 1 | 2.6 | 0.1 | 2.7 | 0.1 | 7.7 |
| DHT Transition 1 | 34.1 | 3.0 | 21.3 | 0.3 | 101.8 |
| DHT Transition 2 | 6.2 | 0.5 | 7.5 | 0.1 | 15.4 |
| C16:0 NAE | 369.2 | 30.8 | 324.4 | 28.7 | 1,524.7 |
| Sphingosine | 41.6 | 3.4 | 36.9 | 0.5 | 168.6 |
| Sphinganine | 6.6 | 0.7 | 2.5 | 0.0 | 24.6 |
| C16:0e MAGE | 238.0 | 11.8 | 250.0 | 21.9 | 428.3 |
| Pregninolone Transition 2 | 6.1 | 0.3 | 6.2 | 0.7 | 15.5 |
| C18:1 NAE | 3.9 | 0.3 | 3.3 | 0.5 | 11.9 |
| C18:0 NAE | 628.3 | 50.8 | 533.0 | 49.0 | 2,559.7 |
| C18:1e MAGE | 804.4 | 108.9 | 271.1 | 11.6 | 4,417.4 |
| C12:0 AC | 1.2 | 0.1 | 1.2 | 0.2 | 6.1 |
| C18:0e MAGE | 300.7 | 31.1 | 194.2 | 11.2 | 1,763.5 |
| C18:2 MAG | 10.9 | 1.4 | 6.1 | 0.3 | 79.1 |
| C18:1 MAG | 53.3 | 5.2 | 38.4 | 0.4 | 256.7 |
| C18:0 MAG | 146.5 | 8.7 | 153.0 | 0.3 | 379.0 |
| Cortisol | 1.9 | 0.4 | 0.4 | 0.1 | 18.6 |
| Cholesteryl Esters | 223,725.8 | 9,159.1 | 230,800.3 | 2,267.2 | 428,537.4 |
| Estradiol Sulfate | 1.3 | 0.1 | 1.4 | 0.0 | 4.0 |
| C20:4 MAG | 1.0 | 0.2 | 0.4 | 0.0 | 9.9 |
| C18:0e/C2:0 MAGE | 33.5 | 4.0 | 13.1 | 0.8 | 184.3 |
| C16:0 AC | 32.5 | 2.5 | 25.8 | 0.1 | 111.4 |
| C22:6 MAG | 0.2 | 0.0 | 0.1 | 0.0 | 2.3 |
| Gamma Tocopherol | 3.9 | 0.2 | 3.6 | 0.0 | 11.6 |
| Pregninolone Sulfate | 865.3 | 85.1 | 757.8 | 354.4 | 8,298.4 |
| C18:0 AC | 9.9 | 0.8 | 9.1 | 0.1 | 40.9 |
| Alpha Tocopherol | 6.3 | 0.5 | 4.6 | 0.6 | 24.7 |
| C16:0e LPEe | 3.4 | 0.1 | 3.5 | 0.2 | 5.7 |
| C16:0 LPE | 4.2 | 1.5 | 0.3 | 0.1 | 124.8 |
| C18:1e LPEe | 0.4 | 0.1 | 0.1 | 0.0 | 5.9 |

| C18:0e LPEe C18:1 LPE C16:0e LPCe C18:0 LPE  C16:0e LPCe  (lysoPAF)  C16:0 LPS C18:0e LPGe C18:1 LPS C18:0 LPS C20:4e LPSe  C16:0 Ceramide C20:4 LPC C20:4 LPS C18:1e LPCe  (lysoPAF) C20:0 LPC  C18:0 Ceramide C16:0/C18:1 DAG C16:0/C20:4 DAG C18:0/C18:1 DAG C18:0/C20:4 DAG C16:0e/C18:1 PEe C16:0 SM  C18:1 SM C18:0 SM  C18:0e/C18:1 PEe C18:0/C18:1 PE C16:0e/C18:1 PCe C16:0e/C18:1 PSe C18:0p/C20:4 PEp C18:0e/C20:4 PEe C20:4 SM C16:0/C18:1 PC C18:0e/C18:1 PGe C16:0p/C20:4 PCp C16:0e/C20:4 PCe C18:0e/C18:1 PCe C16:0/C20:4 PC C16:0/C20:4_PS C18:0/C18:1 PC C18:0p/C20:4 PCp C18:0e/C20:4 PCe C18:0/C20:4 PC C18:0/C20:4 PS | 18.5 | 2.2 | 18.4 | 0.9 | 182.6 |
| --- | --- | --- | --- | --- | --- |
|  | 3.4 | 1.1 | 0.4 | 0.0 | 80.1 |
|  | 65.9 | 4.4 | 60.5 | 0.4 | 203.6 |
|  | 4.2 | 1.0 | 1.6 | 0.0 | 74.5 |
|  | 156.9 | 13.8 | 128.7 | 1.0 | 914.8 |
|  | 4.0 | 0.2 | 3.8 | 0.0 | 11.0 |
|  | 0.7 | 0.1 | 0.3 | 0.0 | 4.2 |
|  | 0.4 | 0.0 | 0.4 | 0.0 | 1.6 |
|  | 0.2 | 0.0 | 0.1 | 0.0 | 2.0 |
|  | 0.1 | 0.0 | 0.0 | 0.0 | 0.9 |
|  | 12.1 | 0.5 | 12.1 | 0.5 | 25.6 |
|  | 307.1 | 15.9 | 296.9 | 3.0 | 737.6 |
|  | 4.1 | 0.1 | 4.1 | 0.1 | 6.6 |
|  | 17.9 | 2.1 | 16.0 | 0.1 | 134.8 |
|  | 15.4 | 1.3 | 13.9 | 0.1 | 85.0 |
|  | 2.0 | 0.1 | 1.8 | 0.0 | 8.5 |
|  | 24.5 | 1.5 | 22.6 | 0.6 | 75.8 |
|  | 1.1 | 0.1 | 1.0 | 0.0 | 3.7 |
|  | 7.7 | 0.5 | 6.8 | 0.2 | 22.2 |
|  | 0.2 | 0.0 | 0.1 | 0.0 | 1.7 |
|  | 0.7 | 0.0 | 0.8 | 0.0 | 1.6 |
|  | 2,210.9 | 68.2 | 2,224.6 | 26.0 | 4,487.0 |
|  | 2,431.0 | 79.3 | 2,436.1 | 35.2 | 5,067.1 |
|  | 2,223.0 | 138.6 | 2,096.8 | 0.6 | 5,354.0 |
|  | 1.3 | 0.3 | 0.8 | 0.1 | 29.9 |
|  | 0.2 | 0.1 | 0.0 | 0.0 | 5.9 |
|  | 2,217.3 | 64.1 | 2,162.4 | 12.8 | 4,441.3 |
|  | 0.0 | 0.0 | 0.0 | 0.0 | 0.8 |
|  | 1.1 | 0.4 | 0.1 | 0.0 | 16.1 |
|  | 0.4 | 0.1 | 0.1 | 0.0 | 4.9 |
|  | 1,431.2 | 72.1 | 1,447.4 | 6.0 | 3,377.9 |
|  | 45,192.5 | 1,323.9 | 46,614.4 | 1,199.4 | 110,129.8 |
|  | 3.4 | 0.8 | 0.4 | 0.0 | 48.4 |
|  | 1,498.1 | 49.6 | 1,454.0 | 21.9 | 2,835.9 |
|  | 2,938.6 | 83.2 | 2,838.1 | 42.1 | 5,398.2 |
|  | 2,453.4 | 83.4 | 2,480.0 | 26.4 | 5,242.7 |
|  | 29,653.5 | 832.0 | 29,010.0 | 702.8 | 60,162.7 |
|  | 0.1 | 0.0 | 0.0 | 0.0 | 1.7 |
|  | 26,727.1 | 824.2 | 27,041.4 | 242.0 | 50,426.4 |
|  | 4,635.6 | 139.7 | 4,569.5 | 45.4 | 8,983.8 |
|  | 4,911.7 | 158.1 | 4,860.5 | 41.5 | 10,835.9 |
|  | 25,456.7 | 722.2 | 25,368.6 | 348.3 | 47,625.3 |
|  | 0.6 | 0.1 | 0.6 | 0.0 | 2.6 |

| C16:0/C16:0/C16:0 TAG | 2,332.2 | 199.4 | 1,756.2 | 74.8 | 8,467.7 |
| --- | --- | --- | --- | --- | --- |
| C16:0/C18:1/C16:0 TAG | 11,485.8 | 614.5 | 10,620.2 | 211.1 | 29,233.4 |
| C16:0/C20:4/C16:0 TAG | 4,208.1 | 179.2 | 3,994.7 | 57.9 | 7,688.3 |
| Lactosylceramide C18:1/C18:0 | 0.7 | 0.1 | 0.5 | 0.1 | 6.7 |
| C18:0/C18:1/C18:0 TAG | 1,213.0 | 107.1 | 898.3 | 5.7 | 4,732.4 |
| C18:0/C18:0/C18:0 TAG | 306.3 | 31.2 | 220.2 | 2.0 | 1,832.1 |
| C18:0/C20:4/C18:0 TAG | 10.3 | 1.9 | 2.8 | 0.0 | 87.4 |

**Supplemental Table 2.** Regression analysis of the targeted plasma metabolomic measures in relation to the concentrations of eleven urine phthalate biomarkers in pregnant women

| **Phthalate Metabolite** | **Metabolite** | **Beta** | **Upper Confidence Interval** | **Lower Confidence Interval** | **P-value** | **N** |
| --- | --- | --- | --- | --- | --- | --- |
| MEP | DHT Transition 1 | 0.37 | 0.55 | 0.18 | 0.0002 | 75 |
| MEP | c18_0_alkyl_lpi | -0.30 | -0.14 | -0.46 | 0.0003 | 87 |
| MEP | C18:1 FFA | 0.17 | 0.27 | 0.06 | 0.0021 | 88 |
| MECPP | c20_4_alkyl_lpi | -0.32 | -0.11 | -0.53 | 0.0034 | 88 |
| MCNP | Testosterone Transition 2 | 0.27 | 0.46 | 0.09 | 0.0047 | 75 |
| MEP | Testosterone Transition 1 | 0.48 | 0.81 | 0.15 | 0.0051 | 75 |
| MEP | C18:1e LPCe (lysoPAF) | 0.31 | 0.53 | 0.09 | 0.0060 | 75 |
| MEHP | N- (phosphonomethyl) glycine 1 | 0.27 | 0.46 | 0.08 | 0.0066 | 91 |
| MCNP | C18:1e LPCe (lysoPAF) | 0.57 | 0.98 | 0.16 | 0.0070 | 75 |
| MEHP | Uridine | 0.25 | 0.43 | 0.07 | 0.0077 | 91 |
| MEOHP | x5_hete | 0.25 | 0.44 | 0.06 | 0.0088 | 84 |
| MEP | C16:0/C20:4/C16:0 TAG | 0.10 | 0.18 | 0.03 | 0.0099 | 75 |
| MEHHP | x5_hete | 0.26 | 0.46 | 0.06 | 0.0104 | 84 |
| MBzP | Arginine | 0.28 | 0.49 | 0.06 | 0.0113 | 91 |
| MEHP | N- (phosphonomethyl) glycine 2 | 0.30 | 0.53 | 0.06 | 0.0129 | 91 |
| MECPP | x5_hete | 0.28 | 0.50 | 0.06 | 0.0140 | 84 |
| MCNP | DHT Transition 1 | 0.46 | 0.83 | 0.10 | 0.0142 | 75 |
| MEP | DHT Transition 2 | 0.28 | 0.50 | 0.06 | 0.0152 | 75 |
| MiBP | C18:0/C18:1 PI | 0.17 | 0.30 | 0.03 | 0.0163 | 88 |
| MEP | C18:0/C18:1/C18:0 TAG | 0.21 | 0.38 | 0.04 | 0.0173 | 75 |
| MEP | Estradiol | 0.12 | 0.21 | 0.02 | 0.0174 | 75 |
| MCNP | C18:0/C18:1 DAG | 0.35 | 0.63 | 0.06 | 0.0177 | 75 |
| MEHP | Asparagine | -0.14 | -0.02 | -0.26 | 0.0184 | 91 |
| MECPP | x18_1_c16_0_ceramide_phos2 | -0.30 | -0.05 | -0.55 | 0.0192 | 88 |
| MBzP | Mevalonic Acid | -0.05 | -0.01 | -0.10 | 0.0192 | 91 |
| MBP | C16:0/C16:0 PI | 0.23 | 0.42 | 0.04 | 0.0195 | 88 |
| MEOHP | c20_4_alkyl_lpi | -0.21 | -0.03 | -0.39 | 0.0198 | 88 |
| MBzP | Adenine | 0.08 | 0.15 | 0.01 | 0.0202 | 91 |
| MEHHP | c06_0_coa | 0.48 | 0.87 | 0.08 | 0.0202 | 48 |
| MEP | C16:0 alkyl LPA | -0.25 | -0.04 | -0.47 | 0.0204 | 87 |
| MiBP | C16:0/C16:0 PI | 0.21 | 0.39 | 0.03 | 0.0205 | 88 |
| MBzP | L-Alanine | 0.35 | 0.64 | 0.05 | 0.0209 | 91 |
| MBzP | L-Aspartic Acid | 0.37 | 0.69 | 0.06 | 0.0210 | 91 |
| MiBP | C16:0/C18:1 PI | 0.16 | 0.30 | 0.02 | 0.0211 | 88 |
| MECPP | C16:0 LPA | -0.28 | -0.04 | -0.52 | 0.0216 | 88 |
| MEP | Cortisol_2 | 0.27 | 0.50 | 0.04 | 0.0226 | 75 |
| MEHHP | c18_1_paf | 0.28 | 0.52 | 0.04 | 0.0228 | 88 |
| MEP | C16:0 FFA | 0.11 | 0.20 | 0.02 | 0.0228 | 88 |
| MEHHP | c20_4_alkyl_lpi | -0.22 | -0.03 | -0.42 | 0.0235 | 88 |
| MCPP | Acetyl-Coa T2 | -0.10 | -0.01 | -0.20 | 0.0266 | 91 |
| MCPP | Deoxyuridine Triphosphate | 0.10 | 0.18 | 0.01 | 0.0278 | 91 |
| MCPP | c20_4_alkyl_lpi | 0.22 | 0.43 | 0.02 | 0.0306 | 88 |
| MCPP | Nitrotyrosine | 0.26 | 0.49 | 0.02 | 0.0307 | 88 |
| MEOHP | c18_1_paf | 0.25 | 0.47 | 0.02 | 0.0309 | 88 |
| MEHHP | x18_1_c16_0_ceramide_phos2 | -0.24 | -0.02 | -0.47 | 0.0338 | 88 |
| MEOHP | c06_0_coa | 0.46 | 0.89 | 0.03 | 0.0348 | 48 |
| MCPP | c22_6_nat | 0.26 | 0.50 | 0.02 | 0.0351 | 88 |
| MEHP | Glutamine | -0.12 | -0.01 | -0.22 | 0.0359 | 91 |
| MECPP | C20:4 LPS | -0.14 | -0.01 | -0.28 | 0.0365 | 75 |
| MEHHP | c22_6_nat | 0.25 | 0.47 | 0.02 | 0.0368 | 88 |
| MEP | Thymine | -0.19 | -0.01 | -0.37 | 0.0372 | 91 |
| MiBP | C18:0/C18:1 alkyl PI | 0.15 | 0.30 | 0.01 | 0.0375 | 88 |
| MEHP | Phenylalanine | -0.20 | -0.01 | -0.39 | 0.0379 | 91 |
| MCPP | C16:0/C16:0 PI | 0.23 | 0.45 | 0.01 | 0.0380 | 88 |
| MBzP | Creatine | 0.14 | 0.28 | 0.01 | 0.0391 | 91 |
| MEHHP | c16_0_acyl_coa | 0.28 | 0.55 | 0.01 | 0.0393 | 67 |
| MBP | DHT Transition 1 | 0.28 | 0.54 | 0.01 | 0.0394 | 75 |
| MEHP | Xanthine | 0.20 | 0.40 | 0.01 | 0.0400 | 91 |
| MCPP | C20:4 LPS | -0.12 | -0.01 | -0.24 | 0.0409 | 75 |
| MiBP | C18:1 FFA | 0.14 | 0.27 | 0.00 | 0.0423 | 88 |
| MEP | pgj2 | -0.15 | 0.00 | -0.29 | 0.0434 | 87 |
| MEP | Hypoxanthine | -0.28 | -0.01 | -0.55 | 0.0435 | 91 |
| MEHP | Tryptophan | -0.12 | 0.00 | -0.23 | 0.0435 | 91 |
| MiBP | C18:0/C20:4 PI | 0.13 | 0.25 | 0.00 | 0.0435 | 88 |
| MiBP | C16:0/C20:4 PI | 0.13 | 0.26 | 0.00 | 0.0435 | 88 |
| MBzP | Histidine | 0.23 | 0.45 | 0.01 | 0.0439 | 91 |
| MBP | C18:1 LPI | 0.18 | 0.35 | 0.00 | 0.0450 | 88 |
| MEOHP | x18_1_c16_0_ceramide_phos2 | -0.21 | 0.00 | -0.42 | 0.0479 | 88 |
| MEP | c22_0_nat | -0.17 | 0.00 | -0.35 | 0.0482 | 88 |
| MEP | Testosterone Transition 2 | 0.10 | 0.21 | 0.00 | 0.0486 | 75 |
| MBzP | c16_0_alkyl_lpi | 0.18 | 0.35 | 0.00 | 0.0486 | 88 |
| MBzP | Serine | 0.25 | 0.50 | 0.00 | 0.0486 | 91 |

**Supplemental Table 3**. Regression analysis of the urine targeted metabolomic measures in relation to the concentrations of eleven urine phthalate biomarkers in pregnant women

| **Phthalate Metabolite** | **Metabolite** | **Beta** | **Upper Confidence Interval** | **Lower Confidence Interval** | **P-value** | **N** |
| --- | --- | --- | --- | --- | --- | --- |
| MiBP | Nicotinamide Mononucleotide | 0.45 | 0.62 | 0.29 | *0.000000332 | 96 |
| MBzP | Nicotinamide Mononucleotide | 0.38 | 0.53 | 0.23 | *0.00000367 | 96 |
| MBP | Nicotinamide Mononucleotide | 0.43 | 0.61 | 0.25 | *0.00000545 | 96 |
| MiBP | Cysteine T2 | 0.32 | 0.46 | 0.18 | *0.0000196 | 96 |
| MCPP | Nicotinamide Mononucleotide | 0.47 | 0.68 | 0.26 | *0.0000256 | 96 |
| MiBP | Cystine | 0.28 | 0.41 | 0.16 | *0.0000258 | 96 |
| MCPP | Cystine | 0.34 | 0.50 | 0.18 | *0.0000368 | 96 |
| MiBP | L-Aspartic Acid | 0.34 | 0.49 | 0.18 | *0.0000451 | 96 |
| MCNP | Nicotinamide Mononucleotide | 0.48 | 0.72 | 0.25 | *0.0001044 | 96 |
| MBP | Cystine | 0.27 | 0.41 | 0.13 | 0.0002 | 96 |
| MBP | Valine | 0.27 | 0.41 | 0.13 | 0.0002 | 96 |
| MiBP | Isoleucine | 0.29 | 0.44 | 0.14 | 0.0002 | 96 |
| MBzP | Valine | 0.23 | 0.35 | 0.11 | 0.0002 | 96 |
| MiBP | Glutamine | 0.30 | 0.46 | 0.15 | 0.0002 | 96 |
| MCPP | Cysteine T2 | 0.34 | 0.52 | 0.16 | 0.0002 | 96 |
| MCPP | Valine | 0.31 | 0.47 | 0.15 | 0.0003 | 96 |
| MBP | Alpha Ketoglutarate | 0.36 | 0.55 | 0.17 | 0.0003 | 95 |
| MBzP | Uridine | 0.27 | 0.42 | 0.13 | 0.0003 | 95 |
| MBP | Cysteine T2 | 0.29 | 0.44 | 0.13 | 0.0004 | 96 |
| MCPP | Alpha Ketoglutarate | 0.40 | 0.62 | 0.18 | 0.0004 | 95 |
| MCPP | Glutamine | 0.36 | 0.55 | 0.16 | 0.0005 | 96 |
| MBP | Glutamine | 0.30 | 0.47 | 0.13 | 0.0006 | 96 |
| MBzP | Isoleucine | 0.25 | 0.39 | 0.11 | 0.0006 | 96 |
| MiBP | Asparagine | 0.25 | 0.38 | 0.11 | 0.0006 | 96 |
| MiBP | Valine | 0.24 | 0.37 | 0.10 | 0.0006 | 96 |
| MBP | L-Aspartic Acid | 0.30 | 0.47 | 0.13 | 0.0007 | 96 |
| MCPP | Glycine T1 | 0.34 | 0.53 | 0.15 | 0.0007 | 96 |
| MBzP | Cystine | 0.21 | 0.33 | 0.09 | 0.0007 | 96 |
| MiBP | Creatine | 0.33 | 0.52 | 0.14 | 0.0009 | 96 |
| MBzP | Glycine T1 | 0.25 | 0.39 | 0.11 | 0.0009 | 96 |
| MBzP | Phenylalanine | 0.21 | 0.34 | 0.09 | 0.0009 | 96 |
| MBP | Nicotinic Acid | 0.27 | 0.43 | 0.11 | 0.0011 | 96 |
| MCNP | N-Acetyl neuraminic acid | 0.55 | 0.88 | 0.23 | 0.0011 | 95 |
| MBzP | Asparagine | 0.21 | 0.34 | 0.09 | 0.0012 | 96 |
| MiBP | 3-Ureidoproprionate | 0.34 | 0.55 | 0.14 | 0.0013 | 96 |
| MCPP | Isoleucine | 0.32 | 0.51 | 0.13 | 0.0013 | 96 |
| MiBP | L-Alanine | 0.31 | 0.49 | 0.12 | 0.0013 | 96 |
| MCPP | L-Aspartic Acid | 0.33 | 0.53 | 0.13 | 0.0013 | 96 |
| MBP | N-Acetyl neuraminic acid | 0.46 | 0.74 | 0.18 | 0.0014 | 95 |
| MCPP | Uridine | 0.32 | 0.52 | 0.13 | 0.0014 | 95 |
| MBP | Thymine T2 | 0.24 | 0.38 | 0.09 | 0.0016 | 96 |
| MiBP | Nicotinic Acid | 0.25 | 0.40 | 0.10 | 0.0017 | 96 |
| MBP | Isoleucine | 0.27 | 0.43 | 0.10 | 0.0017 | 96 |
| MBzP | Nicotinic Acid | 0.23 | 0.36 | 0.09 | 0.0017 | 96 |
| MEHHP | Nicotinamide Mononucleotide | 0.33 | 0.54 | 0.13 | 0.0018 | 96 |
| MEP | Glutamine | 0.24 | 0.39 | 0.09 | 0.0018 | 96 |
| MiBP | Phenylalanine | 0.22 | 0.36 | 0.08 | 0.0018 | 96 |
| MBzP | Tryptophan | 0.23 | 0.38 | 0.09 | 0.0019 | 96 |
| MCNP | Cysteine T2 | 0.32 | 0.52 | 0.12 | 0.0020 | 96 |
| MBzP | Cysteine T2 | 0.22 | 0.35 | 0.08 | 0.0020 | 96 |
| MBzP | Adenine | 0.21 | 0.34 | 0.08 | 0.0021 | 96 |
| MBzP | Creatine | 0.28 | 0.46 | 0.10 | 0.0021 | 96 |
| MCNP | Uridine | 0.32 | 0.52 | 0.12 | 0.0022 | 95 |
| MBzP | L-Aspartic Acid | 0.24 | 0.39 | 0.09 | 0.0022 | 96 |
| MBzP | Alpha Ketoglutarate | 0.26 | 0.42 | 0.09 | 0.0027 | 95 |
| MBzP | Histidine | 0.21 | 0.35 | 0.08 | 0.0028 | 96 |
| MCPP | Nicotinic Acid | 0.29 | 0.47 | 0.10 | 0.0030 | 96 |
| MCNP | Valine | 0.28 | 0.46 | 0.10 | 0.0031 | 96 |
| MBP | Uridine | 0.26 | 0.43 | 0.09 | 0.0033 | 95 |
| MBzP | 3-Ureidoproprionate | 0.29 | 0.48 | 0.10 | 0.0036 | 96 |
| MBzP | Cytosine | 0.17 | 0.28 | 0.06 | 0.0038 | 96 |
| MiBP | Adenine | 0.21 | 0.36 | 0.07 | 0.0039 | 96 |
| MBP | N-Acetyl mannosamine | 0.36 | 0.60 | 0.12 | 0.0041 | 95 |
| MCPP | Asparagine | 0.25 | 0.43 | 0.08 | 0.0042 | 96 |
| MCOP | Glutamine | 0.32 | 0.54 | 0.10 | 0.0042 | 96 |
| MBP | Phenylalanine | 0.21 | 0.36 | 0.07 | 0.0043 | 96 |
| MCPP | Phenylalanine | 0.25 | 0.41 | 0.08 | 0.0043 | 96 |
| MCNP | Glutamine | 0.32 | 0.55 | 0.10 | 0.0043 | 96 |
| MBzP | Pantothenate | 0.25 | 0.42 | 0.08 | 0.0045 | 95 |
| MCOP | Valine | 0.27 | 0.45 | 0.08 | 0.0045 | 96 |
| MCOP | Cysteine T2 | 0.29 | 0.49 | 0.09 | 0.0045 | 96 |
| MiBP | Fumarate | 0.23 | 0.39 | 0.07 | 0.0047 | 96 |
| MCPP | Hypoxanthine | 0.41 | 0.69 | 0.12 | 0.0054 | 95 |
| MCOP | Nicotinamide Mononucleotide | 0.35 | 0.59 | 0.10 | 0.0057 | 96 |
| MBP | Histidine | 0.23 | 0.38 | 0.07 | 0.0060 | 96 |
| MCNP | Nicotinic Acid | 0.29 | 0.50 | 0.09 | 0.0061 | 96 |
| MiBP | Glycine T1 | 0.23 | 0.39 | 0.07 | 0.0062 | 96 |
| MiBP | Thymine T2 | 0.19 | 0.33 | 0.06 | 0.0065 | 96 |
| MCNP | L-Aspartic Acid | 0.31 | 0.54 | 0.09 | 0.0067 | 96 |
| MBzP | L-Alanine | 0.24 | 0.41 | 0.07 | 0.0070 | 96 |
| MBP | Adenine | 0.21 | 0.36 | 0.06 | 0.0070 | 96 |
| MCNP | Thymine T2 | 0.25 | 0.43 | 0.07 | 0.0072 | 96 |
| MCOP | Alpha Ketoglutarate | 0.34 | 0.59 | 0.09 | 0.0073 | 95 |
| MiBP | Cytosine | 0.17 | 0.30 | 0.05 | 0.0075 | 96 |
| MEP | Valine | 0.17 | 0.30 | 0.05 | 0.0075 | 96 |
| MEHP | Nicotinamide Mononucleotide | 0.22 | 0.37 | 0.06 | 0.0076 | 96 |
| MCNP | Thymine T2 | 0.26 | 0.45 | 0.07 | 0.0078 | 96 |
| MBP | Lactic Acid | 0.34 | 0.59 | 0.09 | 0.0083 | 95 |
| MBP | Cytosine | 0.18 | 0.31 | 0.05 | 0.0091 | 96 |
| MBzP | Phosphoenolpyruvic Acid | 0.26 | 0.45 | 0.06 | 0.0095 | 95 |
| MBP | Asparagine | 0.20 | 0.35 | 0.05 | 0.0096 | 96 |
| MCNP | Phenylalanine | 0.25 | 0.44 | 0.06 | 0.0097 | 96 |
| MCOP | Thymine T2 | 0.25 | 0.44 | 0.06 | 0.0101 | 96 |
| MBzP | N-Acetyl mannosamine | 0.28 | 0.49 | 0.07 | 0.0108 | 95 |
| MCOP | Cystine | 0.24 | 0.42 | 0.06 | 0.0108 | 96 |
| MCPP | Cytosine | 0.20 | 0.35 | 0.05 | 0.0110 | 96 |
| MCPP | Histidine | 0.24 | 0.43 | 0.06 | 0.0110 | 96 |
| MCPP | Thymine T2 | 0.21 | 0.38 | 0.05 | 0.0118 | 96 |
| MBP | Pantothenate | 0.26 | 0.46 | 0.06 | 0.0118 | 95 |
| MCNP | Alpha Ketoglutarate | 0.30 | 0.53 | 0.07 | 0.0123 | 95 |
| MBP | Tryptophan | 0.22 | 0.39 | 0.05 | 0.0124 | 96 |
| MCNP | Cystine | 0.23 | 0.42 | 0.05 | 0.0126 | 96 |
| MCPP | Adenine | 0.23 | 0.40 | 0.05 | 0.0132 | 96 |
| MBzP | N-Acetyl neuraminic acid | 0.31 | 0.56 | 0.07 | 0.0132 | 95 |
| MEHHP | Thymine T2 | 0.20 | 0.35 | 0.04 | 0.0133 | 96 |
| MCPP | Creatine | 0.30 | 0.54 | 0.06 | 0.0137 | 96 |
| MEHHP | Nicotinic Acid | 0.23 | 0.40 | 0.05 | 0.0137 | 96 |
| MCNP | N-Acetyl mannosamine | 0.37 | 0.66 | 0.08 | 0.0138 | 95 |
| MCNP | Isoleucine | 0.27 | 0.49 | 0.06 | 0.0141 | 96 |
| MEOHP | Nicotinamide Mononucleotide | 0.25 | 0.46 | 0.05 | 0.0143 | 96 |
| MiBP | Histidine | 0.19 | 0.34 | 0.04 | 0.0144 | 96 |
| MBzP | Adenosine | 0.31 | 0.56 | 0.06 | 0.0150 | 96 |
| MBzP | Uracil | 0.17 | 0.31 | 0.03 | 0.0155 | 96 |
| MBP | Hypoxanthine | 0.31 | 0.55 | 0.06 | 0.0156 | 95 |
| MCNP | Glycine T1 | 0.27 | 0.49 | 0.05 | 0.0156 | 96 |
| MCPP | Thymine T2 | 0.21 | 0.38 | 0.04 | 0.0161 | 96 |
| MCPP | 3-Ureidoproprionate | 0.32 | 0.58 | 0.06 | 0.0165 | 96 |
| MiBP | Tryptophan | 0.20 | 0.36 | 0.04 | 0.0171 | 96 |
| MCOP | Uridine | 0.27 | 0.49 | 0.05 | 0.0177 | 95 |
| MCOP | N-Acetyl neuraminic acid | 0.44 | 0.80 | 0.08 | 0.0181 | 95 |
| MEP | Nicotinamide Mononucleotide | 0.21 | 0.38 | 0.04 | 0.0182 | 96 |
| MBP | Glycine T1 | 0.21 | 0.38 | 0.04 | 0.0185 | 96 |
| MCOP | Thymine T2 | 0.22 | 0.40 | 0.04 | 0.0189 | 96 |
| MCPP | Pantothenate | 0.28 | 0.51 | 0.05 | 0.0194 | 95 |
| MBP | Creatine | 0.25 | 0.46 | 0.04 | 0.0195 | 96 |
| MBP | Adenosine | 0.35 | 0.64 | 0.06 | 0.0196 | 96 |
| MCNP | Cytosine | 0.20 | 0.38 | 0.03 | 0.0198 | 96 |
| MBP | 3-Ureidoproprionate | 0.27 | 0.49 | 0.04 | 0.0203 | 96 |
| MBzP | Hypoxanthine | 0.25 | 0.47 | 0.04 | 0.0215 | 95 |
| MCPP | N-Acetyl mannosamine | 0.34 | 0.62 | 0.05 | 0.0217 | 95 |
| MCNP | Adenine | 0.23 | 0.43 | 0.03 | 0.0232 | 96 |
| MCPP | L-Alanine | 0.27 | 0.50 | 0.04 | 0.0243 | 96 |
| MBzP | Fumarate | 0.17 | 0.32 | 0.02 | 0.0244 | 96 |
| MiBP | Hypoxanthine | 0.27 | 0.50 | 0.04 | 0.0246 | 95 |
| MCPP | Tryptophan | 0.23 | 0.43 | 0.03 | 0.0256 | 96 |
| MEP | Asparagine | 0.15 | 0.29 | 0.02 | 0.0257 | 96 |
| MCOP | Adenine | 0.22 | 0.42 | 0.03 | 0.0259 | 96 |
| MECPP | Thymine T2 | 0.20 | 0.38 | 0.02 | 0.0264 | 96 |
| MCOP | N-Acetyl mannosamine | 0.36 | 0.67 | 0.04 | 0.0271 | 95 |
| MCNP | Asparagine | 0.22 | 0.41 | 0.03 | 0.0272 | 96 |
| MCPP | Citrate | 0.43 | 0.81 | 0.05 | 0.0276 | 95 |
| MCOP | L-Aspartic Acid | 0.25 | 0.48 | 0.03 | 0.0278 | 96 |
| MCNP | Cytidine Monophosphate | 0.35 | 0.66 | 0.04 | 0.0281 | 91 |
| MBzP | Lactic Acid | 0.24 | 0.46 | 0.03 | 0.0293 | 95 |
| MCPP | Fumarate | 0.22 | 0.42 | 0.02 | 0.0297 | 96 |
| MBzP | Thymine T2 | 0.14 | 0.27 | 0.01 | 0.0325 | 96 |
| MCNP | Thymine | 0.28 | 0.53 | 0.02 | 0.0327 | 95 |
| MBzP | Glucose New | 0.29 | 0.56 | 0.02 | 0.0328 | 95 |
| MCNP | Tryptophan | 0.24 | 0.46 | 0.02 | 0.0330 | 96 |
| MBzP | Thymine | 0.20 | 0.39 | 0.02 | 0.0344 | 95 |
| MBzP | Uracil | 0.24 | 0.46 | 0.02 | 0.0344 | 95 |
| MCPP | Phosphoenolpyruvic Acid | 0.28 | 0.54 | 0.02 | 0.0349 | 95 |
| MiBP | Thymine T2 | 0.15 | 0.28 | 0.01 | 0.0352 | 96 |
| MCOP | Nicotinic Acid | 0.23 | 0.44 | 0.02 | 0.0357 | 96 |
| MCOP | Phenylalanine | 0.20 | 0.39 | 0.01 | 0.0357 | 96 |
| MEHP | N-Acetyl neuraminic acid | 0.25 | 0.49 | 0.02 | 0.0363 | 95 |
| MEHHP | Thymine T2 | 0.17 | 0.34 | 0.01 | 0.0368 | 96 |
| MCNP | Histidine | 0.22 | 0.43 | 0.01 | 0.0373 | 96 |
| MBP | Thymine | 0.23 | 0.45 | 0.01 | 0.0373 | 95 |
| MiBP | Uridine | 0.18 | 0.34 | 0.01 | 0.0388 | 95 |
| MCNP | Uracil | 0.22 | 0.43 | 0.01 | 0.0391 | 96 |
| MBP | L-Alanine | 0.21 | 0.41 | 0.01 | 0.0396 | 96 |
| MEHHP | Valine | 0.17 | 0.32 | 0.01 | 0.0413 | 96 |
| MCNP | Phosphoenolpyruvic Acid | 0.27 | 0.54 | 0.01 | 0.0435 | 95 |
| MCNP | Fumarate | 0.22 | 0.44 | 0.01 | 0.0450 | 96 |
| MEP | 3-Ureidoproprionate | 0.20 | 0.40 | 0.00 | 0.0452 | 96 |
| MECPP | Nicotinamide Mononucleotide | 0.25 | 0.49 | 0.01 | 0.0455 | 96 |
| MCPP | Thymine | 0.25 | 0.50 | 0.00 | 0.0466 | 95 |
| MCPP | Glucose New | 0.36 | 0.72 | 0.00 | 0.0475 | 95 |
| MEP | Cysteine T2 | 0.14 | 0.29 | 0.00 | 0.0485 | 96 |
| MEHP | N-Acetyl mannosamine | 0.21 | 0.41 | 0.00 | 0.0490 | 95 |

**Supplemental Table 4.** Relationship of metabolomic compounds and pre-pregnancy BMI.

| **Metabolite** | **Beta** | **Upper Confidence Interval** | **Lower Confidence Interval** | **P-value** | **N** |
| --- | --- | --- | --- | --- | --- |
| Glycine T1 | -1.57 | -0.78 | -2.36 | **0.0002** | 106 |
| Glycine T2 | -1.92 | -0.88 | -2.96 | **0.0004** | 106 |
| Acetylcarnitine | -1.69 | -0.62 | -2.76 | **0.0025** | 106 |
| C16:0/C20:4 PI | -3.87 | -1.28 | -6.45 | **0.0042** | 104 |
| Deoxyuridine Triphosphate | 7.89 | 13.32 | 2.47 | **0.0052** | 106 |
| C16:0/C16:0/C16:0 TAG | -3.38 | -1.01 | -5.75 | **0.0064** | 89 |
| L-Alanine | -1.52 | -0.42 | -2.62 | **0.0077** | 106 |
| Mevalonic Acid | 9.58 | 16.68 | 2.48 | **0.0094** | 106 |
| Isoleucine | -1.93 | -0.49 | -3.37 | **0.0099** | 106 |
| Cholesterol_cholesteryl | -3.04 | -0.77 | -5.32 | **0.0103** | 89 |
| Cysteine T2 | -1.91 | -0.44 | -3.38 | **0.0125** | 106 |
| Histidine | -1.82 | -0.39 | -3.26 | **0.0140** | 106 |
| L-Aspartic Acid | -1.26 | -0.26 | -2.25 | **0.0147** | 106 |
| Valine | -1.88 | -0.35 | -3.40 | **0.0177** | 106 |
| Phenylalanine | -1.93 | -0.35 | -3.51 | **0.0184** | 106 |
| Tryptophan | -3.24 | -0.56 | -5.92 | **0.0195** | 106 |
| Arginine | -1.81 | -0.31 | -3.31 | **0.0197** | 106 |
| Serine | -1.54 | -0.26 | -2.83 | **0.0198** | 106 |
| C16:0/C16:0 PI | -2.30 | -0.39 | -4.22 | **0.0202** | 104 |
| C16:0/C18:1 PI | -2.88 | -0.47 | -5.29 | **0.0212** | 104 |
| C18:0/C20:4 PI | -3.22 | -0.38 | -6.06 | **0.0284** | 104 |
| Proline | -1.52 | -0.17 | -2.87 | **0.0292** | 106 |
| C16:0/C20:4 alkyl PI | 2.17 | 4.21 | 0.13 | **0.0396** | 102 |
| C18:0/C20:4/C18:0 TAG | -1.31 | -0.04 | -2.58 | **0.0459** | 89 |
| Glutamic Acid | -0.73 | 0.00 | -1.45 | 0.0514 | 106 |
| Glutamine | -2.62 | 0.21 | -5.46 | 0.0729 | 106 |
| c14_0_coa | 5.56 | 11.56 | -0.45 | 0.0758 | 50 |
| C18:0/C18:1 PI | -2.32 | 0.22 | -4.85 | 0.0763 | 104 |
| C18:1 alkyl LPI | -1.48 | 0.16 | -3.12 | 0.0795 | 103 |
| C18:0/C20:4 alkyl PI | -2.13 | 0.29 | -4.55 | 0.0868 | 104 |
| c18_1_lpa | 2.25 | 4.80 | -0.30 | 0.0869 | 104 |
| c04_0_coa | 1.79 | 3.82 | -0.25 | 0.0901 | 74 |
| C16:0/C18:1/C16:0 TAG | -2.71 | 0.39 | -5.81 | 0.0905 | 89 |
| Uridine | 1.43 | 3.09 | -0.23 | 0.0947 | 106 |
| Malonyl_coa | -3.17 | 0.53 | -6.86 | 0.0972 | 73 |
| c16_0_18_1_alkyl_pi | -2.05 | 0.37 | -4.46 | 0.1001 | 104 |
| c16_0_paf | 1.76 | 3.88 | -0.37 | 0.1086 | 104 |
| c08_0_coa | -4.48 | 0.94 | -9.90 | 0.1108 | 54 |
| c16_0_coa | 1.99 | 4.45 | -0.46 | 0.1164 | 68 |
| txb2 | 1.76 | 3.97 | -0.44 | 0.1200 | 94 |
| ctp | -2.07 | 0.56 | -4.69 | 0.1254 | 106 |
| Estradiol | 2.91 | 6.66 | -0.84 | 0.1322 | 89 |
| c16_0__alkyl_glycerone_phos | 1.24 | 2.85 | -0.37 | 0.1331 | 104 |
| C18:1 FFA | 2.14 | 4.94 | -0.66 | 0.1379 | 104 |
| Creatine | -1.76 | 0.65 | -4.18 | 0.1554 | 106 |
| C18:1e LPCe  (lysoPAF) | -1.09 | 0.42 | -2.59 | 0.1603 | 89 |
| Lactic Acid | 1.00 | 2.40 | -0.40 | 0.1652 | 106 |
| Testosterone Transition 1 | 0.72 | 1.75 | -0.31 | 0.1734 | 89 |
| C18:0/C18:0/C18:0 TAG | -1.39 | 0.60 | -3.37 | 0.1740 | 89 |
| c16_1_nat | -1.31 | 0.57 | -3.19 | 0.1752 | 103 |
| N-  (phosphonomethyl) glycine 1 | 1.11 | 2.73 | -0.50 | 0.1793 | 106 |
| C20:4 LPS | 2.70 | 6.63 | -1.23 | 0.1809 | 89 |
| 3-Ureidoproprionate | 2.02 | 5.05 | -1.00 | 0.1931 | 106 |
| NADP | -3.60 | 1.86 | -9.06 | 0.1992 | 106 |
| c16_0_18_1_alkyl_pa | 2.70 | 6.83 | -1.43 | 0.2031 | 103 |
| Ribose-15- Biphosphate | -1.92 | 1.03 | -4.86 | 0.2047 | 106 |
| C18:1 alkyl LPA | 1.52 | 3.89 | -0.85 | 0.2122 | 104 |
| C16:0 alkyl LPA | 1.02 | 2.65 | -0.60 | 0.2213 | 103 |
| c20_4_alkyl_lpi | -1.44 | 0.89 | -3.77 | 0.2287 | 104 |
| Pgj2 | 1.39 | 3.66 | -0.88 | 0.2325 | 103 |
| c10_0_coa | -2.13 | 1.34 | -5.60 | 0.2340 | 53 |
| C20:4 FFA | 1.80 | 4.84 | -1.24 | 0.2489 | 103 |
| Pristanic_acid | 1.27 | 3.53 | -1.00 | 0.2763 | 97 |
| Cardiolipin_c18_1 | 1.99 | 5.62 | -1.63 | 0.2843 | 103 |
| Pgf2alpha | 1.05 | 2.97 | -0.88 | 0.2893 | 104 |
| c22_6_nat | 1.11 | 3.19 | -0.97 | 0.2962 | 104 |
| c16_0_alkyl_lpi | 1.04 | 3.05 | -0.97 | 0.3133 | 104 |
| Glucosyl_ceramide | -1.87 | 1.74 | -5.49 | 0.3135 | 74 |
| c16_0_acyl_coa | 0.93 | 2.76 | -0.89 | 0.3184 | 78 |
| c18_0_lpi | 0.98 | 2.93 | -0.97 | 0.3268 | 104 |
| Asparagine | -1.29 | 1.32 | -3.91 | 0.3335 | 106 |
| C16:0 FFA | 1.73 | 5.23 | -1.78 | 0.3367 | 104 |
| Sphinganine | -0.81 | 0.86 | -2.48 | 0.3463 | 89 |
| N-  (phosphonomethyl) glycine 2 | 0.64 | 1.97 | -0.70 | 0.3520 | 106 |
| c06_0_coa | 1.37 | 4.28 | -1.54 | 0.3604 | 61 |
| Adenine | -2.25 | 2.64 | -7.14 | 0.3700 | 106 |
| Guanine | 1.62 | 5.36 | -2.12 | 0.3975 | 106 |
| C18:0/C18:1/C18:0 TAG | -0.90 | 1.17 | -2.97 | 0.3980 | 89 |
| c18_0_paf | 1.04 | 3.44 | -1.37 | 0.3996 | 104 |
| C18:1 LPI | 1.07 | 3.55 | -1.41 | 0.4000 | 104 |
| Cortisol_2 | 0.66 | 2.19 | -0.87 | 0.4004 | 89 |
| c18_1_paf | 0.80 | 2.67 | -1.07 | 0.4032 | 104 |
| C18:0/C18:1 PA | -1.91 | 2.60 | -6.43 | 0.4085 | 103 |
| C18:0/C18:1 alkyl PI | -1.05 | 1.55 | -3.65 | 0.4319 | 104 |
| Coenzyme_a | 1.02 | 3.58 | -1.53 | 0.4337 | 81 |
| C18:0/C20:4 PA | -1.30 | 1.96 | -4.57 | 0.4362 | 103 |
| C18:0 LPA | 1.04 | 3.64 | -1.57 | 0.4372 | 104 |
| c16_0_20_4_alkyl_pa | -0.90 | 1.39 | -3.19 | 0.4421 | 104 |
| x5_hete | 0.92 | 3.28 | -1.44 | 0.4474 | 100 |
| c16_0_c20_4_pa | 0.76 | 2.77 | -1.25 | 0.4613 | 104 |
| C18:0 FFA | 1.56 | 5.74 | -2.61 | 0.4650 | 104 |
| NADPH | -1.85 | 3.14 | -6.84 | 0.4691 | 106 |
| DHT Transition 1 | -0.70 | 1.20 | -2.60 | 0.4716 | 89 |
| Xanthine | 0.59 | 2.21 | -1.03 | 0.4803 | 106 |
| Estradiol Sulfate | 0.48 | 1.94 | -0.98 | 0.5185 | 89 |
| Pge2_pgd2 | 0.76 | 3.09 | -1.56 | 0.5205 | 101 |
| C16:0 LPA | 0.67 | 2.73 | -1.40 | 0.5282 | 104 |
| x_5_6eet | 0.72 | 2.97 | -1.52 | 0.5292 | 104 |
| C20:4 LPA | 0.68 | 2.82 | -1.46 | 0.5325 | 104 |
| c16_0_nat | 0.61 | 2.60 | -1.37 | 0.5461 | 104 |
| Lysine | -0.69 | 1.62 | -2.99 | 0.5610 | 106 |
| Acetyl-CoA T1 | -1.28 | 3.35 | -5.91 | 0.5881 | 106 |
| Acetyl_coa | 0.39 | 1.83 | -1.06 | 0.6000 | 107 |
| Sphingosine | 0.67 | 3.23 | -1.88 | 0.6074 | 89 |
| Phytanic Acid | -0.27 | 0.75 | -1.28 | 0.6092 | 104 |
| DHEA Transition 1 | -0.72 | 2.12 | -3.56 | 0.6218 | 89 |
| C18:0/C20:4 DAG | 0.47 | 2.36 | -1.43 | 0.6295 | 89 |
| c16_0_sphingosine_phosphate | 0.59 | 3.01 | -1.83 | 0.6322 | 103 |
| C16:0/C20:4 DAG | -0.45 | 1.42 | -2.33 | 0.6372 | 89 |
| x15__deoxypgj2 | -0.49 | 1.61 | -2.60 | 0.6452 | 104 |
| c18_1_nat | 0.48 | 2.57 | -1.61 | 0.6538 | 103 |
| C16:0/C18:1 PA | -1.02 | 3.65 | -5.69 | 0.6686 | 103 |
| Hypoxanthine | -0.27 | 0.98 | -1.52 | 0.6719 | 106 |
| Acetyl-Coa T2 | -0.78 | 3.38 | -4.95 | 0.7135 | 106 |
| Cortisol | -0.41 | 1.81 | -2.62 | 0.7201 | 101 |
| DHT Transition 2 | 0.28 | 1.85 | -1.28 | 0.7252 | 89 |
| c12_0_coa | 0.88 | 6.04 | -4.28 | 0.7394 | 42 |
| C18:0/C18:1 alkyl PA | -0.69 | 3.67 | -5.06 | 0.7558 | 103 |
| C18:0/C20:4 alkyl PA | -0.48 | 2.56 | -3.52 | 0.7573 | 104 |
| Adenosine Monophosphate | 0.46 | 3.66 | -2.73 | 0.7777 | 106 |
| DHA | -0.17 | 1.16 | -1.50 | 0.8062 | 104 |
| c22_0_nat | -0.23 | 1.72 | -2.19 | 0.8169 | 104 |
| C18:0/C18:1 DAG | 0.28 | 2.62 | -2.06 | 0.8171 | 89 |
| c20_4_lpi | -0.24 | 1.83 | -2.30 | 0.8221 | 104 |
| c18_0_alkyl_lpi | 0.23 | 2.29 | -1.82 | 0.8244 | 103 |
| Nitrotyrosine | 0.23 | 2.37 | -1.90 | 0.8306 | 104 |
| c20_0_coa | 0.57 | 5.87 | -4.74 | 0.8350 | 46 |
| b-Hydroxyl-b- methylglutaryl-CoA | 0.79 | 9.08 | -7.50 | 0.8524 | 106 |
| C16:0/C20:4/C16:0 TAG | -0.27 | 3.12 | -3.66 | 0.8758 | 89 |
| x18_0_c16_0_ceramide | 0.19 | 3.01 | -2.64 | 0.8976 | 104 |
| c18_2_nat | 0.11 | 2.07 | -1.85 | 0.9157 | 104 |
| BV | -0.08 | 2.05 | -2.21 | 0.9411 | 103 |
| Thymine | 0.06 | 1.89 | -1.77 | 0.9479 | 106 |
| x18_1_c16_0_ceramide_phos2 | 0.04 | 2.07 | -1.98 | 0.9665 | 104 |
| Testosterone Transition 2 | -0.05 | 3.72 | -3.81 | 0.9810 | 89 |
| Inosine | 0.03 | 3.22 | -3.16 | 0.9849 | 106 |
| c16_0_lpi | 0.02 | 2.55 | -2.51 | 0.9862 | 104 |
| DHEA Transition 2 | -0.01 | 1.65 | -1.68 | 0.9888 | 89 |
| C16:0/C18:1 DAG | -0.02 | 2.68 | -2.72 | 0.9907 | 89 |

Bolded p-values are significant, *P*<0.05

BMI=body mass index
